# Binding affinity estimation from restrained umbrella sampling simulations

**DOI:** 10.1038/s43588-022-00389-9

**Published:** 2022-12-29

**Authors:** Vivek Govind Kumar, Adithya Polasa, Shilpi Agrawal, Thallapuranam Krishnaswamy Suresh Kumar, Mahmoud Moradi

**Affiliations:** Department of Chemistry and Biochemistry, University of Arkansas, Fayetteville, AR USA

**Keywords:** Computational biophysics, Biophysical chemistry

## Abstract

The protein–ligand binding affinity quantifies the binding strength between a protein and its ligand. Computer modeling and simulations can be used to estimate the binding affinity or binding free energy using data- or physics-driven methods or a combination thereof. Here we discuss a purely physics-based sampling approach based on biased molecular dynamics simulations. Our proposed method generalizes and simplifies previously suggested stratification strategies that use umbrella sampling or other enhanced sampling simulations with additional collective-variable-based restraints. The approach presented here uses a flexible scheme that can be easily tailored for any system of interest. We estimate the binding affinity of human fibroblast growth factor 1 to heparin hexasaccharide based on the available crystal structure of the complex as the initial model and four different variations of the proposed method to compare against the experimentally determined binding affinity obtained from isothermal titration calorimetry experiments.

## Abstract (web summary)

This work provides a physics-based theoretical framework for accurate protein–ligand binding affinity estimation based on molecular dynamics simulations, enhanced sampling, non-parametric reweighting and the orientation quaternion formalism.

## Main

Accurate quantification of absolute binding affinities remains a problem of major importance in computational biophysics^1–4^. In principle, accurate binding-free-energy calculations should be the cornerstone of any study investigating protein–ligand interactions. However, the high computational costs that typically accompany such calculations necessitate the improvement of the computational methods traditionally used to investigate complex biomolecular interactions^3–5^. Experimentally determined binding affinities are commonly used as benchmarks to judge the accuracy of various computational binding affinity estimation methods^5^. Several experimental techniques can be used to study protein–ligand binding equilibria^5,6^. For instance, isothermal titration calorimetry (ITC) can detect the interaction of binding partners based on changes in solution heat capacity and binding partner concentration^6–8^. Other methods such as fluorescence spectroscopy rely on changes in fluorescence intensity upon ligand binding^6,9,10^. Surface plasmon resonance can be used to calculate binding affinities based on changes in refractive index that occur when an immobilized binding partner interacts with a free binding partner^6^. Studies have found that experimental binding affinities can vary depending on the experimental method used^5^. Therefore, a thorough understanding of the experimental conditions used to generate reference data is essential when comparing computationally determined binding affinities with experimental values.

Several computational methods at varying levels of rigor and complexity have been used to determine binding affinities for biomolecular interactions^3,11–18^. Knowledge-based statistical potentials and force-field scoring potentials are typically used to rank docked protein–ligand or protein–protein complexes but can also be used for binding affinity prediction^19–21^. A major disadvantage of these methods is that they do not treat the entropic effects rigorously, which effectively decreases the accuracy of such binding affinity predictions^5^. This is also the case for methods such as molecular mechanics/Poisson–Boltzmann surface area (MM-PBSA) and molecular mechanics/generalized Born surface area (MM-GBSA), which combine sampling of conformations from explicit solvent molecular dynamics (MD) simulations with free-energy estimation based on implicit continuum solvent models^22–24^. Adequate sampling of protein and ligand conformational dynamics as well as ligand roto-translational movements with respect to the protein is essential for accurately quantifying the entropic reduction arising from the binding event^24–26^. MM-PBSA and MM-GBSA methods typically neglect such entropic contributions to the binding free energy or do not treat them rigorously^23,24^. Binding Free-Energy Estimator 2 (BFEE2) is a state-of-the-art protein–ligand binding affinity calculation software that addresses the substantial shift in configurational enthalpy and entropy that follows ligand–protein binding, which is hard to represent in brute-force simulations^18,27^. An energy–entropy approach, energy–entropy multiscale cell correlation, has been introduced to compute the free energy of binding and has been applied for binding-free-energy calculations, which take into consideration the entropy of all flexible degrees of freedom in the system in a consistent and generic way^28^.

One of the best-known binding-free-energy estimation methods is alchemical free-energy perturbation (FEP), where scaling of non-bonded interactions enables reversal decoupling of the ligand from its environment in the bound state as well as the unbound state^29–32^. Most entropic and enthalpic contributors to changes in binding affinity are typically considered during FEP simulations, thus avoiding the approximations used by methods such as MM-PBSA and MM-GBSA^5,33^. A disadvantage of FEP is the fact that ligands tend to move away from the binding site during the decoupling process, which results in poorly defined target states of the FEP calculation being used as starting states for the re-coupling process^34^. Using receptor–ligand restraints to resolve this issue^30,35–37^ introduces some ambiguity to the way a standard state is defined, with a level of correlation between the size of the simulation cell and the standard state^38^. This can be corrected by the use of appropriate geometrical restraints^39–41^.

Unrestrained long-timescale MD simulations should theoretically allow for the investigation and quantification of protein–ligand or protein–protein binding events^42,43^. While microsecond-level MD simulations provide a more accurate description of protein conformational dynamics compared with shorter simulations^44^, efficient sampling of the conformational landscape remains a major issue and requires access to timescales beyond the capabilities of current MD simulations^45,46^. Several methods have been developed to tackle the sampling problem. Markov state models allow the sampling and characterization of native as well as alternative binding states^57^. Similarly, weighted ensemble simulations sample the conformational landscape along one or more discretized reaction coordinates based on the assignment of a statistical weight to each simulation^47,48^. More traditionally, umbrella sampling (US) along such reaction coordinates can be used to guide the binding or unbinding of a ligand, after which algorithms like the weighted histogram analysis method can be used to calculate a unidimensional potential of mean force (PMF) that quantifies ligand binding and unbinding along a reaction coordinate^49,50^. Better convergence of the calculated free-energy profiles can be achieved by the exchange of conformations between successive US windows as in the bias-exchange umbrella sampling (BEUS)^51–53^. Other methods based on similar principles include umbrella integration^54^, well-tempered metadynamics^55^, adaptive biasing force (ABF) simulations^56^ and variations of these techniques.

Incomplete sampling of important degrees of freedom, such as orientation of the ligand with respect to the protein, remains a major disadvantage of unidimensional PMF-based methods^3,4^. To resolve this problem, ref. 3 reported a method wherein explicitly defined geometrical restraints on the orientation and conformation of the binding partners are used to reduce the conformational entropy of the biomolecular system being studied^3,4^. This results in improved convergence of the PMF calculation^3,4^. The introduction of a restraining potential based on the root-mean-square deviation (RMSD) of the ligand relative to its average bound conformation reduces the flexibility of the ligand and the number of conformations that need to be sampled^3,4^. This method avoids the need to decouple the ligand from its surrounding environment as required by alchemical FEP^3,4,29–32^. Recent studies^4,57^ have described applications and extensions of the methodology proposed by ref. 3.

In this Article, we describe a purely physics-based enhanced sampling method based on biased MD simulations, which is similar in principle to the stratification strategy proposed by refs. ^3,4^. Although we use the US method as our enhanced sampling technique, the methodology is generalizable to other techniques as long as they can be combined with additional restraints. There are several important differences between our method and that of refs. ^3,4^. Our method includes: (1) providing a general scheme that can be easily adapted to any number of restraints; (2) the non-parametric reconstruction of the grid PMF, as defined below; and (3) the use of the unidimensional orientation angle of the ligand with respect to the protein as a collective variable for restraining, as opposed to the use of three Euler angles. We note that the method of refs. ^3,4^ can in principle be generalized as well; the generalization is not as straightforward as it is in our proposed method, particularly in removing some of the restraints. In other words, while adding more restraints is somewhat similar to our approach in the method of of refs. ^3,4^, removing some of the restraint requires less trivial changes to the formalism that makes it distinct from our method. We have used this methodology to calculate the binding affinity for the interaction of human fibroblast growth factor 1 (hFGF1) with heparin hexasaccharide, its glycosaminoglycan (GAG) binding partner. hFGF1 is an important signaling protein that is implicated in physiological processes such as cell proliferation and differentiation, neurogenesis, wound healing, tumor growth and angiogenesis^58–62^. GAGs are linear anionic polysaccharides that interact with positively charged regions of FGF binding partners to regulate their biological activity^63^. The hFGF1–heparin complex is the most well-known and broadly characterized protein–GAG complex^64,65^. Heparin binding is thought to stabilize hFGF1 and impart protection against proteolytic degradation. In this study, we show that the absolute binding affinity for the hFGF1–heparin interaction calculated using our approach is in good agreement with binding affinity data from ITC experiments. Four alternative methods are used for estimating the absolute binding affinity within the formalism presented here to determine the workings of the methodology and the effect of the application of different (or no) restraints. We also compare our results with those obtained from FEP simulations and show that although performing longer FEP simulations could improve the accuracy of binding affinity estimates when compared with short FEP simulations, our approach is still more accurate than FEP when similar simulation times are used.

## Results

### Calculation of binding free energy using four different strategies

We have calculated the absolute binding free energy for the interaction of hFGF1 with heparin hexasaccharide using four variations of the stratification scheme described above, based on a combination of steered MD (SMD) and BEUS simulations. The details of the methodology are discussed in Methods. Four different methods are used with varying effectiveness in estimating the absolute binding free energy. These methods are: (1) the traditional distance-based BEUS simulations that do not employ any additional restraining; (2) distance-based BEUS simulations employing a restraint on the orientation of the ligand (*Ω*) defined based on the orientation quaternion formalism; (3) distance-based BEUS simulations employing a restraint on the RMSD of both ligand and protein (*r*_L_ and *r*_P_); (4) distance-based BEUS simulations employing a restraint on the RMSD of both ligand and protein as well as the orientation of the ligand (*Ω*, *r*_L_ and *r*_P_). In each case, appropriate correction terms are calculated as discussed in the ‘Theoretical foundation’ section and shown in Table 1.

**Table 1:** Summary of the results of free-energy calculations

Quantity	No restraints	*Ω* restraint	*r*_L_ and *r*_P_ restraint	*Ω*, *r*_L_ and *r*_P_ restraint
Grid PMF difference (kcal mol^−1^)	Δ*G*(**x**_B_) = −19.7 ± 1.1^a^	Δ*G*_*Ω*_(**x**_B_) = −13.2 ± 0.3	${{\Delta }}G_{r_{\mathrm{L}},r_{\mathrm{P}}}( {{{{\boldsymbol{x}}}}_{\mathrm{B}}})$ = −17.7 ± 1.0	${{\Delta }}G_{{\varOmega},{r_{\mathrm{L}},r_{\mathrm{P}}}}( {{{{\boldsymbol{x}}}}_{\mathrm{B}}})$ = −17.0 ± 0.5
Orientation correction (kcal mol^−1^)	NA	Δ*U*_*Ω*_(**x**_B_) = 4.4 ± 0.3	NA	${{\Delta }}U_{{\varOmega},{r_{\mathrm{L}},r_{\mathrm{P}}}}( {{{{\boldsymbol{x}}}}_{\mathrm{B}}})$ = 4.6 ± 0.3
Ligand RMSD correction (kcal mol^−1^)	NA	N/A	${{\Delta }}U_{r_{\mathrm{L}}}( {{{{\boldsymbol{x}}}}_{\mathrm{B}}})$ = 0.6 ± 0.1	${{\Delta }}U_{r_{\mathrm{L}}}( {{{{\boldsymbol{x}}}}_{\mathrm{B}}})$ = 0.6 ± 0.1
Protein RMSD correction (kcal mol^−1^)	NA	NA	${{\Delta }}U_{{{r}}_{{{\mathrm{P}}}}}^{r_{\mathrm{L}}}\left( {{{{\boldsymbol{x}}}}_{\mathrm{B}}} \right)$ = 0.3 ± 0.1	${{\Delta }}U_{{{r}}_{{{\mathrm{P}}}}}^{r_{\mathrm{L}}}\left( {{{{\boldsymbol{x}}}}_{\mathrm{B}}} \right)$ = 0.3 ± 0.1
Δ*G*_V_ (kcal mol^−1^)	3.7 ± 0.2	2.5 ± 0.2	2.3 ± 0.2	2.7 ± 0.2
Δ*G*°(kcal mol^−1^)	−16.0 ± 1.2	−6.3 ± 0.5	−14.5 ± 1.0	−8.7 ± 0.7
*K*_d_ (μM)^b^	*O*(10^−6^)	25	*O*(10^−5^)	0.5
*K*_d_ range (μM)^c^	10^−7^–10^−5^	11–58	10^−6^–10^−4^	0.2−2.0

We are comparing the Δ*G*° of four different restraining methods (see ‘BEUS simulations’ in Methods for details).

^a^All error estimates are based on 1 s.d.

^b^Equilibrium dissociation constant (*K*_d_) values are determined directly from mean absolute binding-free-energy (Δ*G*°) values using relation (2).

^c^*K*_d_ range is determined from the lower and upper limits of Δ*G*° values (mean ± s.d.) using relation (2). The experimentally determined *K*_d_ and Δ*G*° were 1.68 ± 0.03 μM and −7.88 ± 0.01 kcal mol^−1^, respectively (see Fig. 4). The orientation angle of heparin with respect to the protein (*Ω*), RMSD of the protein (*r*_P_), RMSD of heparin (*r*_L_), and the contribution of the difference between the volume of the binding pocket and the bulk to the binding free energy (Δ*G*_V_). Δ*G*(**x**_B_), Δ*G*_*Ω*_(**x**_B_), ${{\Delta }}G_{r_{\mathrm{L}},r_{\mathrm{P}}}\left( {{{{\boldsymbol{x}}}}_{\mathrm{B}}} \right)$ and ${{\Delta }}G_{{{\varOmega }},r_{\mathrm{L}},r_{\mathrm{P}}}\left( {{{{\boldsymbol{x}}}}_{\mathrm{B}}} \right)$ are the PMF difference between the binding pocket center and the bulk associated with respective restraints. NA means the data are not applicable in the corresponding section. Δ*U*_*Ω*_(**x**_B_), ${{\Delta }}U_{{\varOmega }}^{r_{\mathrm{L}},r_{\mathrm{P}}}\left( {{{{\boldsymbol{x}}}}_{\mathrm{B}}} \right)$, ${{\Delta }}U_{{{r}}_{{{\mathrm{L}}}}}\left( {{{{\boldsymbol{x}}}}_{\mathrm{B}}} \right)$ and ${{\Delta }}U_{{{r}}_{{{\mathrm{P}}}}}^{r_{\mathrm{L}}}\left( {{{{\boldsymbol{x}}}}_{\mathrm{B}}} \right)$ are correction terms associated with restraints (see ‘Theoretical foundation’ in Methods for details).

**Fig. 1:**
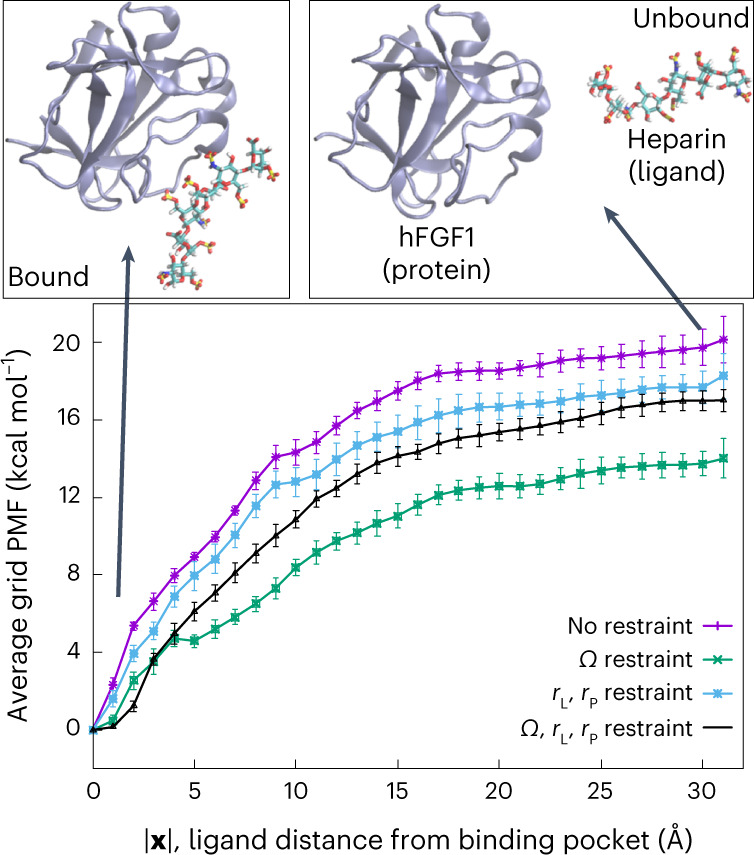
Average grid PMF based on our alternative distance-based BEUS simulations. Average grid PMFs in terms of |**x**|, where **x** is the three-dimensional position vector of the ligand with respect to the center of the binding pocket. The *x* axis represents |**x**| and the *y* axis represents Δ*F*(|**x**|), which is an average over all Δ*F*(**x**) with the same |**x**|, that is, the ligand distance from the center of the binding pocket. The error bars represent the standard deviation obtained from all values of Δ*F*(**x**) at various grid points **x** with the same |**x**|. Representative images of heparin hexasaccharide bound to hFGF1 (|**x**| = 0 Å) and unbound (|**x**| = 30 Å) are shown above the grid PMF plot. We used *n* = 31 MD replicas to generate the data in all cases. Source data

**Fig. 2:**
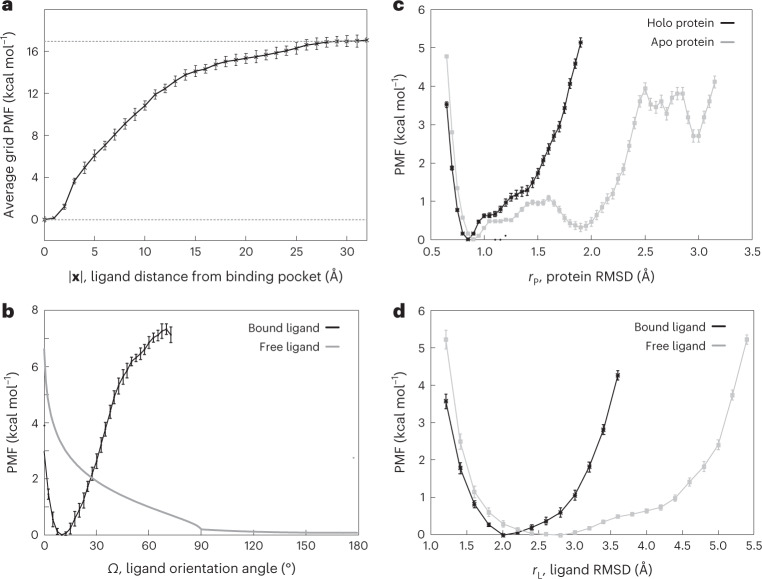
Computational heparin–hFGF1 binding-free-energy measurements. **a**, Average grid PMF in terms of |**x**|, where **x** is the three-dimensional position vector of the ligand with respect to the center of the binding pocket determined from distance-based BEUS simulations with *Ω*, *r*_L_ and *r*_P_ restraints. The *x* axis represents |**x**| and the *y* axis represents ${{\Delta }}G_{{{\varOmega }},r_{\mathrm{L}},r_{\mathrm{P}}}\left( {\left| {{{\boldsymbol{x}}}} \right|} \right)$, which is an average over all ${{\Delta }}G_{{{\varOmega }},r_{\mathrm{L}},r_{\mathrm{P}}}\left( {{{\boldsymbol{x}}}} \right)$ with the same |**x**|, that is, the ligand distance from the center of the binding pocket. The error bars represents the standard deviation obtained from all values of ${{\Delta }}G_{{{\varOmega }},r_{\mathrm{L}},r_{\mathrm{P}}}\left( {{{\boldsymbol{x}}}} \right)$ at various grid points **x** with the same |**x**|. The dashed line represents the value associated with ${{\Delta }}G_{{{\varOmega }},r_{\mathrm{L}},r_{\mathrm{P}}}\left( {|{{{\boldsymbol{x}}}}|} \right)$ at |**x**| = 30 Å. **b**, The PMF associated with the ligand orientation angle (*Ω*) for the bound heparin (that is, **x** ≈ **0**, ligand in the binding pocket) and free heparin (that is, **x** ≈ **x**_B_, ligand in the bulk). The latter is calculated analytically with the help of relation (21). **c**,**d**, PMF in terms of internal conformational fluctuations of the protein and ligand. **c**, PMF associated with the internal RMSD of heparin-bound (black line) and apo (gray line) hFGF1 (*r*_P_). **d**, PMF associated with the internal RMSD of FGF1-bound (black line) and free (gray line) heparin hexasaccharide (*r*_L_). The error bars in **b**–**d** represent the standard deviation determined from the bootstrapping algorithm described in Methods. We used *n* = 31, *n* = 30 and *n* = 12 MD replicas to generate the data shown in **a**, **b**, and **c** and **d**, respectively. Source data

We denote the PMF of the ligand at a given position **x** (with respect to the center of the heparin binding pocket) as the grid PMF, as the PMF is estimated at different grid points in this approach (Fig. 1). The average grid PMF profiles along the ligand–protein distance for the four different methods used here (as shown in Fig. 1) confirm the differential behavior of these methods (see Supplementary Fig. 1 for a schematic representation of these simulations). Note that since **x** = **0** is the grid point associated with the lowest PMF by definition, the average PMF along |**x**| has its global minimum at |**x**| = 0. The most successful method is expected to be the one employing restraints on *Ω*, *r*_L_ and *r*_P_ (Table 1 and Fig. 1). The largest contributor to the free energy is the difference between the grid PMF associated with the heparin hexasaccharide at a grid point at the center of the binding pocket and at any grid point in the bulk, which is −17.0 ± 0.5 kcal mol^−1^ (Fig. 2a and Table 1).

The PMF calculations above are based on the BEUS simulations along the protein–ligand distance; however, the orientation and RMSD of the ligand and the RMSD of the protein are restrained to speed up convergence. To account for the orientation bias, a correction term needs to be applied, which is calculated from the PMF associated with the ligand orientation angle at the bulk and binding pocket (Fig. 2b). The orientation bias is estimated to be 4.4 ± 0.3 kcal mol^−1^ (Table 1). Similarly, a correction term is calculated based on the PMF of the ligand RMSD and that of the protein (Fig. 2c). These correction terms are estimated to be 0.7 ± 0.1 kcal mol^−1^ and 0.4 ± 0.1 kcal mol^−1^ for the ligand and protein, respectively (Table 1).

**Fig. 3:**
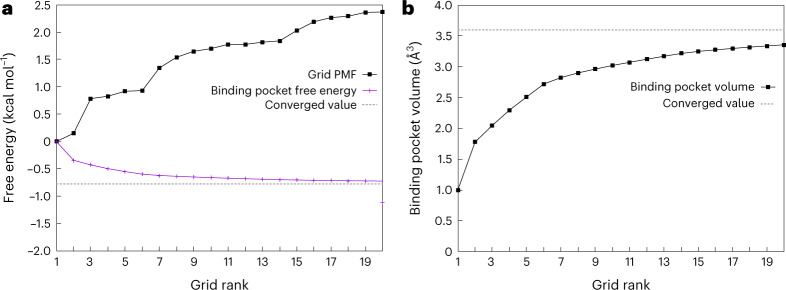
Estimating binding pocket volume and its contribution to absolute binding free energy. **a**, Grid PMF (Δ*G*(**x**)) associated with grid points with the 20-lowest PMF values (black) along with estimated Δ*G*_P_ based on these grid points (shown in an accumulative manner in magenta). The distance-based BEUS simulations with no restraints are used for these calculations. The dashed line shows the estimated Δ*G*_P_ based on all visited grid points inside or outside the binding pocket. The *x* axis shows the ranking of these 20 grid points from lowest PMF onwards. **b**, Binding pocket volume (*V*_P_) calculated from the 20-lowest grid PMF values (similar to **a**). The dashed line shows the *V*_P_ estimated from all visited grid points inside or outside the binding pocket. See ‘Theoretical foundation’ in Methods for more details. Source data

Finally, another term is needed to account for the difference in the volume accessible to the ligand in the binding pocket and in the bulk (volume contribution). Figure 3 shows that the binding pocket contribution (Δ*G*_P_) (or binding pocket volume (*V*_P_)) for the distance-based BEUS simulations with no restraint as determined from the 20-lowest free-energy grid points is almost equal to that obtained from all visited grid points inside or outside the binding pocket. For the distance-based BEUS simulations with *Ω*, *r*_L_ and *r*_P_ restraints, this term is estimated to be 2.7 ± 0.2 kcal mol^−1^ (Table 1), which results in an absolute binding free energy of −8.7 ± 0.7 kcal mol^−1^ (Table 1).

**Fig. 4:**
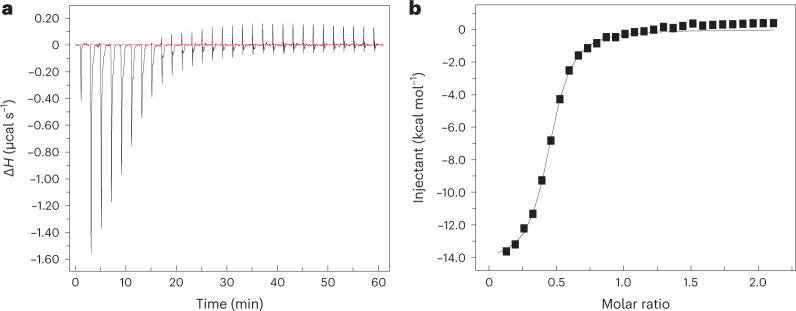
Experimental heparin–hFGF1 binding-free-energy measurements. Isothermogram representing the titration of hFGF1 with heparin hexasaccharide. **a**, The raw data. The horizontal red line represents the zero axis in the plot. **b**, The best fit of the raw data (one set of sites binding model). In the specific experiment shown, *K*_d_ is 1.68 ± 0.03 μM and the free energy from experimental *K*_d_ is −7.88 ± 0.01 kcal mol^−1^. Δ*H*, heat change. Source data

On the basis of our error analysis, equilibrium dissociation constant (*K*_d_) values calculated from the absolute binding free energy were found to be in the micromolar range with an average value of 0.6 μM (using the mean absolute binding free energy (Δ*G*°) estimate) and ranging from 0.2 μM to 2.0 μM (based on the lower and upper bounds of free energy estimates) (Table 1). These are in very good agreement with the *K*_d_ value obtained from ITC experiments. We performed the ITC experiments in triplicate resulting in a *K*_d_ of 1.68 ± 0.03 μM (as shown in Fig. 4), 1.65 ± 0.07 μM and 1.69 ± 0.05 μM in three independent experiments. The binding free energy calculated from the experimental *K*_d_ (−7.87 kcal mol^−1^, −7.88 kcal mol^−1^ or −7.89 kcal mol^−1^, depending on the experiment) is also in good agreement with the computationally calculated binding free energy (Fig. 4 and Table 1).

### Comparison between computationally and experimentally calculated binding free energy of heparin–hFGF1

**Fig. 5:**
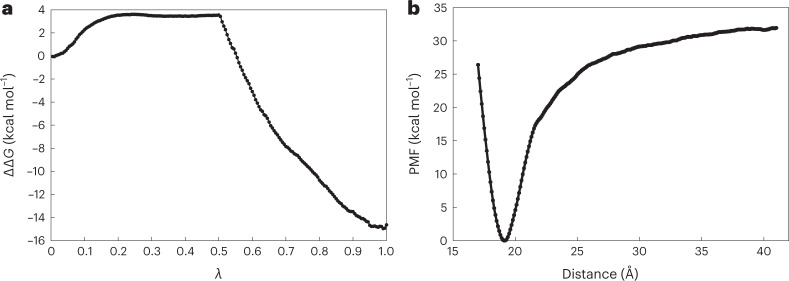
Binding-free-energy measurements using BFEE2 software. **a**, Free-energy difference for the double annihilation of heparin hexasaccharide in its bound state and in water from FEP calculations. *λ* is a coupling parameter that gradually switches on interactions between a fully coupled state (*λ* = 0) and a final, fully decoupled state (*λ* = 1). **b**, PMF obtained during the separation of hFGF1 and heparin hexasaccharide using the geometric route. We used the BFEE2 (ref. 27) package to estimate the absolute free energy of binding in silico for an alchemical or geometrical route with multiple subprocesses and geometric constraints. The alchemical and geometric methods’ binding-free-energy estimates were 0.55 ± 30.25 kcal mol^−1^ and −19.04 ± 2.95 kcal mol^−1^, respectively. See Supplementary Tables 2 and 3 for details of each geometric restriction and alchemical FEP calculations, respectively. Source data

The quantitative agreement between the computational and experimental binding affinity estimates is a great indicator of the accuracy of our absolute binding-free-energy calculation method. However, if proper restraining is not used as in the distance-based BEUS simulations with no restraints or only RMSD restraints, the binding affinity estimates would be off by several orders of magnitude. The simulations that restrain only the orientation of the ligand are interestingly quite successful as well, being off by only one order of magnitude in terms of binding affinity, which is generally considered a good estimate. This provides some evidence that the orientation of the ligand is perhaps the degree of freedom with the most substantial contribution to the absolute binding free energy besides the ligand–protein distance. While the average grid PMF profiles along the ligand–protein distance (as shown in Fig. 1) confirm that the four methods used here produce different results, it is important to note that the correction terms should ideally eliminate these differences. This is seen to some extent when comparing the two methods involving orientation restraints that happen to estimate binding affinities that are reasonably close (Table 1) to the experimentally determined value. Another source of error in our calculations could be in estimating the *V*_P_ and eventually the contribution of the difference between the volume of the binding pocket and the bulk to the binding free energy (Δ*G*_V_). In doing so, we have made an assumption that the *V*_P_ can be calculated from relation (31) approximating the grid PMF with that obtained from biased simulations. Comparing Δ*G*_V_ values from Table 1 shows that different biases result in different approximating values ranging from 2.3 kcal mol^−1^ to 3.7 kcal mol^−1^. For more information on these results and the convergence of data, see Supplementary Table 1 and Figs. 2–5 ref. 27.

### Examining how this approach compares with other prevalent binding-free-energy calculation methods

Recent computational studies have used the MM-GBSA method to calculate the binding free energy of the hFGF1–heparin interaction, with values ranging from −84.2 kcal mol^−1^ to −106.1 kcal mol^−1^ (ref. 66). The outcomes of the MM-GBSA technique are considerably different from those of ours. The MM-PBSA and MM-GBSA, which is not an all-atom simulation approach like ours, has drawbacks including the continuum solvent approximation. The intrinsic dielectric constant’s appropriate setting presents another challenge. It has long been known that the selection of the intrinsic dielectric constant has a significant impact on the computed electrostatic energy^22–24^. However, these contributors to the binding affinity are typically taken into account during FEP simulations, thus obviating the need for the approximations used in MM-PBSA and MM-GBSA^5,33^. It is widely accepted that binding-free-energy estimates from MM-PBSA and MM-GBSA are less accurate than those from FEP, which is considered to be the gold standard for the calculation of absolute binding affinities^67^. We performed double annihilation FEP to calculate the absolute binding free energy of the hFGF1–heparin complex. The BFEE2 method was used to estimate binding affinities from FEP simulations with the consideration of several restraints to improve sampling within the framework of the method of refs. ^3,4^. The FEP simulations here were designed to have an aggregate simulation time comparable to that used in our BEUS simulations (~2.3 μs for FEP compared with ~1.1 μs for SMD + BEUS). An absolute binding free energy of 0.55 ± 30.25 kcal mol^−1^ was obtained for the FEP (Fig. 5a and Supplementary Table 2). Unlike the absolute binding free energy estimated from the BEUS simulations (−8.7 ± 0.7 kcal mol^−1^) (Table 1), the estimates from the FEP simulations are not in a good agreement with the binding free energy determined from ITC experiments (−7.88 ± 0.01 kcal mol^−1^) (Fig. 4). More importantly, a large uncertainty is associated with the FEP results that is due to the relative large size of the ligand. To show the effectiveness of our plan, we also calculated the binding free energies of hFGF1 and heparin directly using the method of refs. ^3,4^ as implemented within the BFEE2 package (the geometrical route). To make a fair comparison, we ran 1.6 μs of aggregate simulation using the ABF free-energy calculations. Heparin and hFGF1 have a binding free energy of −19.04 ± 2.95 kcal mol^−1^ based on the geometric approach (Fig. 5b and Supplementary Table 3). In contrast to our technique, the BFEE2 geometric route anticipated a value that was twice as high as the experimental value of free energy. This comparison shows the effectiveness of our method over well-established binding-free-energy calculation methods. To firmly establish the efficiency of our strategy, additional research with a bigger data sample will be required in the future. In particular, it is important to determine what parameters make our method more efficient than the BFEE2 geometric protocol. For instance, it could be due to use of BEUS simulation scheme or a more fundamental difference regarding the use of simpler restraints and analysis schemes.

Studies have shown that the binding affinity and free-energy results derived from computational methods can be compared with experimental binding affinities obtained from ITC experiments^7,8^. However, for a reliable computational free-energy estimate, employing purely physics-based free-energy calculation methods such as those employed here has proven to be difficult. Herein we showed that using a careful strategy that considers all relevant free-energy terms and ensures the use of powerful enhanced sampling techniques could result in good quantitative agreements between the computational and experimental binding affinity estimates. Our methodology could serve as a robust free-energy calculation method for determining the binding affinities of any protein and ligand of interest. However, the accuracy of the resulting binding free energies is still limited by the reliability of the force field parameters, which is at least equally as important as sampling for accurate physics-based binding affinity estimation.

## Discussion

The formalism presented in this work has notable similarities to the method previously proposed by ref. 3, and later implemented^4,57^. However, there are major differences that make the current method more practical. The grid PMF and its various estimates provide a simple conceptual framework to understand how restraining can be accounted for with appropriate correction terms. The average grid PMF in terms of the ligand–protein distance provides an alternative to the PMF in terms of *d* as is often constructed. The non-parametric reweighting allows for calculating the grid PMF in terms of the distance from the center of the binding pocket, as defined in this work, eliminating the need for calculating the PMF in terms of the polar and azimuthal angles as in the method of refs. ^3,4^. Relation (30) is a general scheme that can be easily adapted to any number of restraints. For instance, one may or may not add the polar and azimuthal angles to the restraints using the trivial generalization of relation (30). The orientation angle of the ligand with respect to the protein as determined using the orientation quaternion formalism provides a simple way of determining the absolute binding free energy with a feasible computational cost. Among the four different sets of restraints, the two involving orientation restraints predict binding free energies similar to that determined experimentally. Again, if restraining the orientation angle does not allow for a rapid convergence, one can add more restraints including the tilt and/or spin restraints. While the traces of the method of refs. ^3,4^ is clear in our derivation of the binding free energy, there are also clear differences in the use of the concept of the grid PMF that allows treating any restraints within the general formalism expressed in relation (30). A more extensive work is needed to determine when restraints in addition to those used in this work are necessary.

The outcomes of a simulation are significantly influenced by a variety of other factors, including model quality and the precision of docking. The efficiency of the method’s findings may also be impacted by the degree to which the force field of ligands is accurately modeled. The restrictions that are often associated with these kinds of approach are connected to sampling, which might vary from project to project; for instance, bigger ligands may demand greater sampling than smaller ligands do. Also extremely crucial is the beginning structure of the bound state; the more precise the bound state, the more accurate the binding affinities will be.

## Methods

### Theoretical foundation

Binding affinity is often quantified using the equilibrium dissociation constant (*K*_d_), defined as:

1 $$K_{\mathrm{d}} = \left[ {\mathrm{P}} \right]\left[ {\mathrm{L}} \right]/\left[ {{\mathrm{P}}:{\mathrm{L}}} \right]$$

where [P], [L] and [P:L] are the concentrations of protein, ligand and the protein–ligand complex, respectively. Computationally, the absolute binding free energy (Δ*G*°), which is the standard molar free energy of binding, is more convenient to calculate. The dissociation constant and the absolute binding free energy are related via

2 $$\Delta G^\circ = RT\ln \frac{{K_{\mathrm{d}}}}{{1\,{\mathrm{M}}}}$$

where *R* is the gas constant, *T* is the temperature and 1 M is 1 molar concentration. Various strategies have been used to estimate Δ*G*°, some of which were briefly discussed above. The methodology proposed here has a notable resemblance to the stratification strategy of refs. ^3,4^. However, the two methods have major differences as will be discussed later in this section.

Absolute binding free energy or Δ*G*° is the free-energy change associated with moving the ligand from the bulk to the binding pocket (Supplementary Table 1). Within the formalism presented in this work, Δ*G*° is determined from the grid PMF *G*(**x**), where **x** is the position of the ligand mass center from the center of the binding pocket (Supplementary Table 1), *G*(**x**) is the PMF associated with the ligand position **x**. In practice, we need to bin the three-dimensional space and define the PMF at every bin or grid point as:

3 $$G\left( {{{\boldsymbol{x}}}} \right) = - RT\ln p\left( {{{\boldsymbol{x}}}} \right)$$

where *p*(**x**) is the probability of finding the ligand at bin **x**.

We define Δ*G*(**x**) = *G*(**x**) − *G*(**0**), where **x** = **0** (that is, the center of the binding pocket) is defined as the grid point associated with the lowest grid PMF. One can show:

4 $$\Delta G^\circ = - RT\ln \frac{{{\int}_{{\mathrm{pocket}}} {{\mathrm{e}}^{ - \frac{{G\left( {{{\boldsymbol{x}}}} \right)}}{{RT}}}} {\mathrm{d}}V}}{{{\int}_{{\mathrm{bulk}}} {{\mathrm{e}}^{ - \frac{{G\left( {{{\boldsymbol{x}}}} \right)}}{{RT}}}} {\mathrm{d}}V}} = - RT\ln \frac{{{\int}_{{\mathrm{pocket}}} {{\mathrm{e}}^{ - \frac{{{{\Delta }}G\left( {{{\boldsymbol{x}}}} \right)}}{{RT}}}} {\mathrm{d}}V}}{{{\int}_{{\mathrm{bulk}}} {{\mathrm{e}}^{ - \frac{{{{\Delta }}G\left( {{{\boldsymbol{x}}}} \right)}}{{RT}}}} {\mathrm{d}}V}}$$

in which the binding ‘pocket’ refers to all **x** ∈ **V** where the ligand is considered bound and ‘bulk’ refers to all **x** ∈ **V** where the ligand is not interacting with the protein. **V** here is a subset of space with a single protein in standard concentration (that is, 1 M). As Δ*G*(**x**) is the same everywhere in the bulk, we can simplify relation (4) as follows:

5 $$\Delta G^\circ = - RT\ln \frac{{V_{\mathrm{P}}}}{{{\mathrm{e}}^{ - \frac{{{{\Delta }}G\left( {{{{\boldsymbol{x}}}}_{\mathrm{B}}} \right)}}{{RT}}}V_{\mathrm{B}}}} = - {{\Delta }}G\left( {{{{\boldsymbol{x}}}}_{\mathrm{B}}} \right) - RT\ln \frac{{V_{\mathrm{P}}}}{{V_{\mathrm{B}}}}$$

where *V*_B_ is the bulk volume per protein associated with the standard concentration, **x**_B_ is any grid point in the bulk and *V*_P_ is the binding pocket volume defined as:

6 $$V_{\mathrm{P}} = \mathop {\int}\nolimits_{{\mathrm{pocket}}} {{\mathrm{e}}^{ - \frac{{{{\Delta }}G\left( {{{\boldsymbol{x}}}} \right)}}{{RT}}}} {\mathrm{d}}V$$

Defining Δ*G*_V_ as the contribution of the difference between the volume of the binding pocket and the bulk to the binding free energy:

7 $${{\Delta }}G_{\mathrm{V}} = - RT\ln \frac{{V_{\mathrm{P}}}}{{V_{\mathrm{B}}}}$$

Combining equations (5) and (7), we have:

8 $$\Delta G^\circ = - {{\Delta }}G\left( {{{{\boldsymbol{x}}}}_{\mathrm{B}}} \right) + {{\Delta }}G_{\mathrm{V}}$$

We can find the bulk volume (*V*_B_) associated with the standard concentration for a single protein approximately as:

9 $$V_{\mathrm{B}} = \frac{{\frac{1}{{N_{\mathrm{A}}}}\,{\mathrm{mol}}}}{{1\,{\mathrm{M}}}} = \frac{1}{{N_{\mathrm{A}}}}{\mathrm{L}} \approx 1,661\,{{\text{\AA}}}^3$$

where *N*_A_ is Avogadro’s constant and L is the unit of volume (litres). We can now rewrite Δ*G*_V_ as:

10 $$\Delta G_{\mathrm{V}} = - RT\ln \frac{{V_{\mathrm{P}}}}{{V_{\mathrm{B}}}} = - RT\ln \frac{{V_{\mathrm{P}}}}{{{{\text{\AA}}}^3}} + RT\ln \frac{{V_{\mathrm{B}}}}{{{{\text{\AA}}}^3}} = \Delta G_{\mathrm{P}} - \Delta G_{\mathrm{B}}$$

in which Δ*G*_B_ is the bulk volume contribution and Δ*G*_P_ is the binding pocket contribution:

11 $$\left\{ {\begin{array}{*{20}{l}} {\Delta G_{\mathrm{B}} = - RT\ln \frac{{V_{\mathrm{B}}}}{{{{\text{\AA}}}^3}} \approx - 7.42RT} \hfill \\ {\Delta G_{\mathrm{P}} = - RT\ln \frac{{V_{\mathrm{P}}}}{{{{\text{\AA}}}^3}} = - RT\ln \mathop {\int}\nolimits_{{\mathrm{pocket}}} {{\mathrm{e}}^{ - \frac{{{{\Delta }}G\left( {{{\boldsymbol{x}}}} \right)}}{{RT}}}} \frac{{{\mathrm{d}}V}}{{{{\text{\AA}}}^3}}} \hfill \end{array}} \right.$$

Determining both Δ*G*(**x**_B_) and Δ*G*_P_ requires finding the grid PMF Δ*G*(**x**). Δ*G*(**x**_B_) is the PMF difference between the binding pocket center and the bulk and Δ*G*_P_ also requires an estimate for Δ*G*(**x**) within the binding pocket. We therefore do not need to find Δ*G*(**x**) for all **x** if we have a good estimate for Δ*G*(**x**) within the binding pocket and in the bulk. Ideally, Δ*G*(**x**) for these points can be determined by pulling the ligand out of the binding pocket towards the bulk and using an enhanced sampling technique such as US to sample the space of a collective variable such as *d*, that is, the distance between the mass centers of the ligand and protein. Δ*G*(**x**) can be estimated for all sampled grid points **x** using this distance-based US simulation. Note that the collective variable used for biasing would be *d*, while the collective variable used for the PMF calculations would be the three-dimensional position vector of the mass center of ligand with respect to protein’s binding pocket center. One may estimate the grid PMF from the distance-based US simulations using a non-parametric reweighting algorithm as discussed in this section. Δ*G*(**x**) can also be used to estimate Δ*G*_P_ as defined in relation (11). There is often no need to strictly define the binding pocket as only low Δ*G*(**x**) values have non-negligible contribution to *V*_P_ and thus even if we include all sampled grid points, only those close to the binding pocket center have non-negligible contributions.

A practical issue with determining Δ*G*(**x**_B_) is the convergence. The key obstacles for the sampling that slow down the convergence are the orientation of the ligand, and the conformational changes of the ligand and protein. Using an approach similar in spirit to the previously proposed stratification strategy^3,4,24^, we can circumvent extensive sampling of these degrees of freedom. Let us first focus on the orientation of the ligand (*Ω*), defined using the orientation quaternion formalism. We can restrain *Ω* during the distance-based US simulations using a biasing potential ($\frac{1}{2}k{{\varOmega }}^2$ where a *k* is harmonic force constant) and later correct the free-energy difference based on the PMF associated with the *Ω*, which is different in the bulk (*F*(**x**_B_, *Ω*)) and in the binding pocket (*F*(**0**, *Ω*)). More generally, for any grid point **x**, we may determine Δ*G*(**x**) based on the PMF associated with the *Ω* at **x** (*F*(**x**, *Ω*)) and **0** (*F*(**0**, *Ω*)):

12 $${\mathrm{e}}^{ - \frac{{\Delta G({{{\boldsymbol{x}}}})}}{{RT}}} = \frac{{{\int}_{\!0}^\uppi {{\mathrm{e}}^{ - \frac{{F({{{\boldsymbol{x}}}},\varOmega )}}{{RT}}}{\mathrm{d}}\varOmega } }}{{{\int}_{\!0}^\uppi {{\mathrm{e}}^{ - \frac{{F(\boldsymbol{0},\varOmega )}}{{RT}}}{\mathrm{d}}\varOmega } }}$$

Note that *F*(**x**, *Ω*) is the PMF associated with **x** and *Ω*, defined such that:

13 $$G{{{\mathrm{(}}}}{{{\boldsymbol{x}}}}{{{\mathrm{)}}}} = c - RT\ln \mathop {\int}\nolimits_{\!0}^\uppi {{\mathrm{e}}^{ - \frac{{F({{{\boldsymbol{x}}}},\varOmega )}}{{RT}}}{\mathrm{d}}\varOmega }$$

where *c* is an arbitrary constant. We therefore have:

14 $${\mathrm{e}}^{ - \frac{{\Delta G({{{\boldsymbol{x}}}})}}{{RT}}} = \frac{{{\int}_{\!0}^\uppi {{\mathrm{e}}^{ - \frac{{F({{{\boldsymbol{x}}}},\varOmega )}}{{RT}}}{\mathrm{d}}\varOmega } }}{{{\int}_{\!0}^\uppi {{\mathrm{e}}^{ - \frac{{F({{{\boldsymbol{x}}}},\varOmega ) + \frac{1}{2}k\varOmega ^2}}{{RT}}}{\mathrm{d}}\varOmega } }} \times \frac{{{\int}_{\!0}^\uppi {e^{ - \frac{{F({\boldsymbol{0}},\varOmega ) + \frac{1}{2}k\varOmega ^2}}{{RT}}}{\mathrm{d}}\varOmega } }}{{{\int}_{\!0}^\uppi {e^{ - \frac{{F({\boldsymbol{0}},\varOmega )}}{{RT}}}{\mathrm{d}}\varOmega } }} \times \frac{{{\int}_{\!0}^\uppi {{\mathrm{e}}^{ - \frac{{F({{{\boldsymbol{x}}}},\varOmega ) + \frac{1}{2}k\varOmega ^2}}{{RT}}}{\mathrm{d}}\varOmega } }}{{{\int}_{\!0}^\uppi {{\mathrm{e}}^{ - \frac{{F({\boldsymbol{0}},\varOmega ) + \frac{1}{2}k\varOmega ^2}}{{RT}}}{\mathrm{d}}\varOmega } }}$$

We now define *G*_*Ω*_(**x**) as the grid PMF of the restrained system (by *Ω*):

15 $$G_\varOmega ({{{\boldsymbol{x}}}}) = - RT\ln \mathop {\int}\nolimits_{\!0}^\uppi {{\mathrm{e}}^{ - \frac{{F({{{\boldsymbol{x}}}},\varOmega ) + \frac{1}{2}k\varOmega ^2}}{{RT}}}{\mathrm{d}}\varOmega }$$

We also define *U*_Ω_(**x**) as the average biasing potential at grid point **x**:

16 $$U_{{\varOmega }}\left( {{{\boldsymbol{x}}}} \right) = - RT\ln \langle {{\mathrm{e}}^{ - \frac{{\frac{1}{2}k{{\varOmega }}^2}}{{RT}}}} \rangle _{{{\boldsymbol{x}}}} = - RT\ln \frac{{{\int}_0^\uppi {{\mathrm{e}}^{ - \frac{{F\left( {{{{\boldsymbol{x}}}},{{\varOmega }}} \right) + \frac{1}{2}k{{\varOmega }}^2}}{{RT}}}} {\mathrm{d}}{{\varOmega }}}}{{{\int}_0^\uppi {e^{ - \frac{{F\left( {{{{\boldsymbol{x}}}},{{\varOmega }}} \right)}}{{RT}}}} {\mathrm{d}}{{\varOmega }}}}$$

Now we have from relations (14), (15) and (16):

17 $${{\Delta }}G\left( {{{\boldsymbol{x}}}} \right) = {{\Delta }}G_{{\varOmega }}\left( {{{\boldsymbol{x}}}} \right) - {{\Delta }}U_{{\varOmega }}\left( {{{\boldsymbol{x}}}} \right)$$

where, the free energy of grid point **x** from the center **0** (Δ*G*(**x**)) is calculated based on its equivalent free energy (Δ*G*_Ω_(**x**)) in a system biased by a harmonic restraint on *Ω* and a correction term Δ*U*_Ω_(**x**). For **x** = **x**_B_:

18 $${{\Delta }}U_{{\varOmega }}\left( {{{{\boldsymbol{x}}}}_{\mathrm{B}}} \right) = - RT\ln \frac{{\langle {{\mathrm{e}}^{ - \frac{{\frac{1}{2}k\varOmega ^2}}{{RT}}}} \rangle _{{\mathrm{bulk}}}}}{{\langle {{\mathrm{e}}^{ - \frac{{\frac{1}{2}k\varOmega ^2}}{{RT}}}} \rangle _{{\mathrm{pocket}}}}}$$

To determine the above ensemble averages, we need to determine the PMF along *Ω* for the bound and unbound ligand and calculate the ensemble averages analytically using relation (16). Δ*G*_*Ω*_(**x**_B_) can be determined from PMF calculations, where the distance between the protein and ligand is varied and the orientation of the ligand is restrained (distance-based BEUS with restrained orientation). We note that:

19 $$V_{\mathrm{P}} = \mathop {\int}\nolimits_{{\mathrm{pocket}}} {{\mathrm{e}}^{ - \frac{{{{\Delta }}G\left( {{{\boldsymbol{x}}}} \right)}}{{RT}}}} {\mathrm{d}}V = \mathop {\int}\nolimits_{{\mathrm{pocket}}} {{\mathrm{e}}^{ - \frac{{{{\Delta }}G_{{\varOmega }}\left( {{{\boldsymbol{x}}}} \right) - {{\Delta }}U_{{\varOmega }}\left( {{{\boldsymbol{x}}}} \right)}}{{RT}}}} {\mathrm{d}}V \approx \mathop {\int}\nolimits_{{\mathrm{pocket}}} {{\mathrm{e}}^{ - \frac{{{{\Delta }}G_{{\varOmega }}\left( {{{\boldsymbol{x}}}} \right)}}{{RT}}}} {\mathrm{d}}V$$

where we assume Δ*U*_*Ω*_(**x**) is negligible for **x** within the binding pocket. In other words, $\langle {{\mathrm{e}}^{ - \frac{{\frac{1}{2}k{{\varOmega }}^2}}{{RT}}}} \rangle _{{{\boldsymbol{x}}}} \approx \langle {{\mathrm{e}}^{ - \frac{{\frac{1}{2}k{{\varOmega }}^2}}{{RT}}}} \rangle _{\boldsymbol{0}}$ for **x** close to **0**.

In brief, if we choose to restrain the orientation, our absolute binding-free-energy estimate includes the following terms (using relations (8) and (17)):

20 $$\Delta G^\circ = - \Delta G_\varOmega \left( {{{{\boldsymbol{x}}}}_{\mathrm{B}}} \right) + \Delta {{U}}_\varOmega \left( {{{{\boldsymbol{x}}}}_{\mathrm{B}}} \right) + \Delta G_{\mathrm{V}}$$

*F*(**x**_B_, *Ω*) can be calculated numerically from orientation angle distribution of a free ligand: ${{F}}({{{\boldsymbol{x}}}}_{\mathrm{B}},{{\varOmega }}) = - RT\ln p\left( {{\varOmega }} \right),$ where *p*(*Ω*) is determined from the distribution of Euler angles ($p\left( {\phi ,\theta ,\psi } \right) = \frac{1}{{8\uppi ^2}}\sin \theta$, where 0 ≤ *ϕ*, *ψ* ≤ 2π and 0 ≤ *θ* ≤ π) given that:

21 $$\cos \frac{{\varOmega }}{2} = \cos \frac{\phi }{2}\cos \frac{\theta }{2}\cos \frac{\psi }{2} + \sin \frac{\phi }{2}\sin \frac{\theta }{2}\sin \frac{\psi }{2}$$

$\langle {{\mathrm{e}}^{ - \frac{{\frac{1}{2}k{{\varOmega }}^2}}{{RT}}}} \rangle _{{\mathrm{bulk}}}$ can then be calculated using relation (16) with numerically estimated *F*(**x**_B_, *Ω*) and the *k* value used in the simulations. $F\left( {{{{\boldsymbol{x}}}}_{\mathrm{B}},{{\varOmega }}} \right) = - RT\ln p({{\varOmega }})$ was numerically estimated by discretizing each of the 3 Euler angles with a bin width of 1° and a total of 360 **×** 360 **×** 180 bins to estimate *p*(*Ω*) from *p*(*ϕ*, *θ*, *ψ*). *F*(**0**, *Ω*) can be determined approximately using orientation-based US simulations of bound ligand. *F*(**0**, *Ω*) can then be used to estimate $\langle {{\mathrm{e}}^{ - \frac{{\frac{1}{2}k{{\varOmega }}^2}}{{RT}}}} \rangle _{{\mathrm{pocket}}}$ using relation (16).

The above strategy can be extended to other degrees of freedom for which unbiased sampling may hinder the convergence. Most notably, the internal conformational changes of the ligand and that of the protein may also play a crucial role in slowing down the convergence. In the following, we show how one can restrain not only the orientation of the ligand but also the RMSD of the ligand (denoted here by *r*) in distance-based US simulations (along *d*) to speed up convergence. In this case, the grid PMF difference Δ*G*(**x**) is calculated based on Δ*G*_*Ω*,*r*_(**x**), the grid PMF of a system whose *Ω* and *r* are both restrained:

22 $${\mathrm{e}}^{ - \frac{{\Delta G\left( {\boldsymbol{x}} \right)}}{{RT}}} = \frac{{{\int}_{\!0}^\infty {{\int}_0^\uppi {{\mathrm{e}}^{ - \frac{{F\left( {{\boldsymbol{x}},\varOmega ,r} \right)}}{{RT}}}} } {\mathrm{d}}\varOmega {\mathrm{d}}r}}{{{\int}_{\!0}^\infty {{\int}_{\!0}^\uppi {{\mathrm{e}}^{ - \frac{{F\left( {{\boldsymbol{0}},\varOmega ,r} \right)}}{{RT}}}} } {\mathrm{d}}\varOmega {\mathrm{d}}r}}$$

Using a similar strategy as in relation (14), we have:

23 $$\begin{array}{l}{\mathrm{e}}^{ - \frac{{\Delta G\left( x \right)}}{{RT}}} = \frac{{\mathop {\smallint }\nolimits_0^\infty \mathop {\smallint }\nolimits_0^\uppi {\mathrm{e}}^{ - \frac{{F\left( {{\boldsymbol{x}},\Omega ,r} \right)}}{{RT}}}{\mathrm{d}}\varOmega {\mathrm{d}}r}}{{\mathop {\smallint }\nolimits_0^\infty \mathop {\smallint }\nolimits_0^\uppi {\mathrm{e}}^{ - \frac{{F\left( {{\boldsymbol{x}},\varOmega ,r} \right) + \frac{1}{2}k^\prime r^2}}{{RT}}}{\mathrm{d}}\varOmega {\mathrm{d}}r}} \times \frac{{\mathop {\smallint }\nolimits_0^\infty \mathop {\smallint }\nolimits_0^\uppi {\mathrm{e}}^{ - \frac{{F\left( {{\boldsymbol{x}},\varOmega ,r} \right) + \frac{1}{2}k^\prime r^2}}{{RT}}}{\mathrm{d}}\varOmega {\mathrm{d}}r}}{{\mathop {\smallint }\nolimits_0^\infty \mathop {\smallint }\nolimits_0^\uppi {\mathrm{e}}^{ - \frac{{F\left( {{\boldsymbol{x}},\varOmega ,r} \right) + \frac{1}{2}k^\prime r^2 + \frac{1}{2}k\varOmega ^2}}{{RT}}}{\mathrm{d}}\varOmega {\mathrm{d}}r}}\\ \times \frac{{\mathop {\smallint }\nolimits_0^\infty \mathop {\smallint }\nolimits_0^\uppi {\mathrm{e}}^{ - \frac{{F\left( {0,\varOmega ,r} \right) + \frac{1}{2}k^\prime r^2 + \frac{1}{2}k\varOmega ^2}}{{RT}}}{\mathrm{d}}\varOmega {\mathrm{d}}r}}{{\mathop {\smallint }\nolimits_0^\infty \mathop {\smallint }\nolimits_0^\uppi {\mathrm{e}}^{ - \frac{{F\left( {{\boldsymbol{0}},\varOmega ,r} \right) + \frac{1}{2}k^\prime r^2}}{{RT}}}{\mathrm{d}}\varOmega {\mathrm{d}}r}} \times \frac{{\mathop {\smallint }\nolimits_0^\infty \mathop {\smallint }\nolimits_0^\uppi {\mathrm{e}}^{ - \frac{{F\left( {{\boldsymbol{0}},\varOmega ,r} \right) + \frac{1}{2}k^\prime r^2}}{{RT}}}{\mathrm{d}}\varOmega {\mathrm{d}}r}}{{\mathop {\smallint }\nolimits_0^\infty \mathop {\smallint }\nolimits_0^\uppi {\mathrm{e}}^{ - \frac{{F\left( {{\boldsymbol{0}},\varOmega ,r} \right)}}{{RT}}}{\mathrm{d}}\varOmega {\mathrm{d}}r}}\\ \times \frac{{\mathop {\smallint }\nolimits_0^\infty \mathop {\smallint }\nolimits_0^\uppi {\mathrm{e}}^{ - \frac{{F\left( {{\boldsymbol{x}},\varOmega ,r} \right) + \frac{1}{2}k^\prime r^2 + \frac{1}{2}k\varOmega ^2}}{{RT}}}{\mathrm{d}}\varOmega {\mathrm{d}}r}}{{\mathop {\smallint }\nolimits_0^\infty \mathop {\smallint }\nolimits_0^\uppi {\mathrm{e}}^{ - \frac{{F\left( {{\boldsymbol{0}},\varOmega ,r} \right) + \frac{1}{2}k^\prime r^2 + \frac{1}{2}k\varOmega ^2}}{{RT}}}{\mathrm{d}}\varOmega {\mathrm{d}}r}}\end{array}$$

which results in:

24 $${{\rm{e}}^{ - \frac{{\Delta G\left( {\bf{x}} \right)}}{{RT}}}} = \frac{{{{\langle {{\rm{e}}^{ - \frac{{\frac{1}{2}{k^\prime }{r^2}}}{{RT}}}}\rangle }_{\bf{0}}}}}{{{{\langle {{\rm{e}}^{ - \frac{{\frac{1}{2}{k^\prime }{r^2}}}{{RT}}}}\rangle }_{\bf{x}}}}} \times \frac{{{{\langle {{\rm{e}}^{ - \frac{{\frac{1}{2}k{\varOmega ^2}}}{{RT}}}}\rangle }_{}}{0^r}}}{{\langle {{\rm{e}}^{ - \frac{{\frac{1}{2}k{\varOmega ^2}}}{{RT}}}}\rangle _{\bf{x}}^r}} \times \frac{{{{\rm{e}}^{ - \beta {G_{\varOmega ,r}}({\bf{x}})}}}}{{{{\rm{e}}^{ - \beta {G_{\varOmega ,r}}({\bf{0}})}}}}$$

Here we have defined *G*_*Ω*,*r*_(**x**) as:

25 $$G_{{{\varOmega }},{{r}}}\left( {{{\boldsymbol{x}}}} \right) = - RT\ln \mathop {\int}\nolimits_0^\infty {\mathop {\int}\nolimits_0^\uppi {{\mathrm{e}}^{ - \frac{{F\left( {{{{\boldsymbol{x}}}},{{\varOmega }},{{r}}} \right) + \frac{1}{2}k^\prime {{r}}^2 + \frac{1}{2}k{{\varOmega }}^2}}{{RT}}}} }$$

where *k*′ is the harmonic force constant associated with the *r* based on biasing potential ($\frac{1}{2}k^\prime {{r}}^2$). We also define *U*_*r*_(**x**) similar to *U*_*Ω*_(**x**) in relation (15) except for using *r* instead of *Ω*. $U_{\varOmega}^{r}({\boldsymbol{x}})$ is also defined similar to *U*_Ω_(***x***) except for the additional restraint on *r*:

26 $$U_\varOmega ^r\left( x \right) = - RT\ln \langle {{\mathrm{e}}^{ - \frac{{\frac{1}{2}k\varOmega ^2}}{{RT}}}} \rangle _{\boldsymbol{x}}^r = - RT\ln \frac{{\mathop {\smallint }\nolimits_0^\infty \mathop {\smallint }\nolimits_0^\uppi {\mathrm{e}}^{ - \frac{{F\left( {{\boldsymbol{x}},\varOmega ,r} \right) + \frac{1}{2}k^\prime r^2 + \frac{1}{2}k\varOmega ^2}}{{RT}}}{\mathrm{d}}\varOmega {\mathrm{d}}r}}{{\mathop {\smallint }\nolimits_0^\infty \mathop {\smallint }\nolimits_0^\uppi {\mathrm{e}}^{ - \frac{{\left( {F\left( {{\boldsymbol{x}},\varOmega ,r} \right) + \frac{1}{2}k^\prime r^2} \right)}}{{RT}}}{\mathrm{d}}\varOmega {\mathrm{d}}r}}$$

Finally, we have:

27 $${{\Delta }}G\left( {{{\boldsymbol{x}}}} \right) = {{\Delta }}G_{{{\varOmega }},{{r}}}\left( {{{\boldsymbol{x}}}} \right) - {{\Delta }}U_{{r}}\left( {{{\boldsymbol{x}}}} \right) - {{\Delta }}U_{{\varOmega }}^r\left( {{{\boldsymbol{x}}}} \right)$$

In brief, if we choose to restrain both the orientation and RMSD, our absolute binding-free-energy estimate includes the following terms:

28 $$\Delta G^\circ = - \Delta G_{\varOmega ,r}\left( {{{{\boldsymbol{x}}}}_{\mathrm{B}}} \right) + \Delta U_r\left( {{{{\boldsymbol{x}}}}_{\mathrm{B}}} \right) + \Delta U_\varOmega ^r\left( {{{{\boldsymbol{x}}}}_{\mathrm{B}}} \right) + \Delta G_{\mathrm{V}}$$

Here we are using an approximation similar to that in relation (19):

29 $$V_{\mathrm{P}} \approx \mathop {\int}\nolimits_{{\mathrm{pocket}}} {{\mathrm{e}}^{ - \frac{{{{\Delta }}G_{{{\varOmega }},{{r}}}\left( {{{\boldsymbol{x}}}} \right)}}{{RT}}}} {\mathrm{d}}V$$

Using relations (20) and (28), we can generalize the stratification strategy to include three restraints on arbitrary collective variables *α*, *β* and *γ*:

30 $$\Delta G^\circ = - \Delta G_{\alpha ,\beta ,\gamma }\left( {{{{\boldsymbol{x}}}}_{\mathrm{B}}} \right) + \Delta {{U}}_\gamma \left( {{{{\boldsymbol{x}}}}_{\mathrm{B}}} \right) + \Delta {{U}}_\beta ^\gamma \left( {{{{\boldsymbol{x}}}}_{\mathrm{B}}} \right) + \Delta {{U}}_\alpha ^{\beta ,\gamma }\left( {{{{\boldsymbol{x}}}}_{\mathrm{B}}} \right) + \Delta G_{\mathrm{V}}$$

where:

31 $$\Delta G_{\mathrm{V}} \approx - RT\ln \mathop {\int}\nolimits_{{\mathrm{pocket}}} {{\mathrm{e}}^{ - \frac{{\Delta G_{\alpha ,\beta ,\gamma }({{{\boldsymbol{x}}}})}}{{RT}}}} \frac{{{\mathrm{d}}V}}{{{{\text{\AA}}}^3}} - \Delta G_{\mathrm{B}}$$

### Isothermal titration calorimetry of hFGF1 with heparin hexasaccharide

ITC data were obtained using MicroCal iTC 200 (Malvern) with microcal origin software. The change in heat during the biomolecular interaction was measured by titrating heparin (loaded in the syringe) into the hFGF1 solution in the calorimetric cell. Both the protein and the heparin samples were prepared in the buffer containing 10 mM phosphate buffer with 100 mM NaCl at pH 7.2 and were degassed before loading. The protein-to-heparin ratio was maintained at 1:10 with the protein concentration being 100 μM and the heparin concentration being 1 mM. A total of 30 injections were conducted with a constant temperature of 25 °C and stirring speed of 300 rpm. One set of sites binding model was used for the ITC binding curve^68^. The standard binding free energy Δ*G*° was determined from dissociation constant via relation (2) at *T* = 25 °C. The experiment was repeated three times with the same sample and the results obtained were very similar to each other. The mean and standard deviation were reported for both *K*_d_ and Δ*G*°.

### All-atom MD simulations

For our bound state, we utilized the X-ray crystal structure of the dimeric hFGF1 combination with heparin hexasaccharide (PDB 2AXM; resolution, 3.0 Å)^69^, and for our apo state, we used the X-ray crystal structure of monomeric hFGF1 (PDB 1RG8; resolution, 1.1 Å)^70^. The NAMD 2.13 (ref. 71) was used to run MD simulations. Using a conjugate gradient, we energy-minimized the system for 10,000 steps. We next relaxed the systems using stepwise restrained MD simulations (for 1 ns) using CHARMM-GUI^72^. All production runs were done in an NPT (constant N, number of atoms; P, pressure; T, temperature) ensemble after the first NVT (constant N, number of atoms; V, volume; T, temperature) relaxation. Simulations were done at 300 K with a 2 fs time step and a 0.5 ps^−1^ damping coefficient using a Langevin integrator. Nosé–Hoover–Langevin pistons were used to maintain 1 atm pressure^72^. Long-range electrostatic interactions were estimated using the particle mesh Ewald approach. The initial runs were done for 15 ns, followed by the production run on the Anton 2 supercomputer (Pittsburgh Supercomputing Center) for 4.8 μs with a 2.5 fs time step.

### MD simulations of free heparin hexasaccharide

Heparin hexasaccharide^69^ was simulated in a rectangular water box without the protein. The system was set up as described previously in the ‘All-atom MD simulations’ section. The final conformation after relaxation was then used as the starting conformation for 10 production runs for 40 ns each. The total simulation time was around 400 ns.

### SMD simulations

The final conformations of the hFGF1–heparin^73^, apo hFGF1 (ref. 73) and free heparin hexasaccharide equilibrium simulations were used to generate starting conformations for the non-equilibrium pulling simulations. Four collective variables^74^ were used for the SMD simulations^75^: (1) distance between the heavy-atom center of mass of heparin and that of the protein (*d*); (2) the orientation angle of heparin with respect to the protein (*Ω*) defined using the orientation quanternion formalism; (3) RMSD of the protein (*r*_P_); (4) RMSD of heparin (*r*_L_). Six independent sets of simulations were performed. The distance-based SMD simulation was run for 9.5 ns, while the orientation-based SMD simulation was run for 8 ns. The distance-based SMD simulation was used to pull the heparin away from the protein by approximately 30 Å (10 Å **→** 40 Å) with a force constant of 100 kcal (mol Å^2^)^−1^. The orientation angle was also restrained in this simulation with a force constant of 0.5 kcal (mol degree^2^)^−1^ to stay close to its initial orientation in the bound state. The orientation-based SMD simulation was used to rotate the bound heparin locally with respect to the protein (0° → 73°) with a force constant of 100 kcal (mol degree^2^)^−1^. Four RMSD-based SMD simulations were run for 10 ns each using a force constant of 50 kcal (mol Å^2^)^−1^: (1) to change the RMSD of the bound protein (0.5 Å→2 Å) (the RMSD of heparin was restrained in this simulation with a force constant of 1 kcal (mol Å^2^)^−1^); (2) to change the RMSD of the bound heparin (1.5 Å → 4 Å); (3) to change the RMSD of the unbound protein (0.8 Å → 3.2 Å); (4) to change the RMSD of the free heparin (1.5 Å → 5.5 Å).

### BEUS simulations

BEUS^53,76,77^, which is a variation of the US simulation method, was performed to estimate grid PMF (Supplementary Fig. 1). Four independent sets of distance (*d*)-based BEUS simulations were performed, with no restraints, restraint on orientation angle of heparin with respect to the protein (*Ω*), restraint on RMSD of the ligand (*r*_L_) and RMSD of the protein (*r*_P_), and restraints on *Ω*, *r*_L_ and *r*_P_. Two sets of BEUS simulations were also performed using the *Ω* collective variable, one with and one without a restraint on *r*_L_ and *r*_P_. In addition, two sets of BEUS simulations were performed using the *r*_P_ collective variable (bound protein with restraint on *r*_L_; unbound protein) and two sets were performed using the *r*_L_ collective variable (bound ligand; free ligand). Selected SMD conformations were assigned to individual BEUS windows with equal spacing in each one of these BEUS simulations. The distance-based BEUS simulations ran for 10 ns with 31 replicas/windows and the orientation-based simulations ran for 10 ns with 30 replicas/windows. The RMSD-based BEUS simulations ran for 10 ns with 12 replicas/windows. The force constant used for ligand–protein distance (*d*) in distance-based BEUS was 2 kcal (mol Å^2^)^−1^ while the orientation was restrained as in SMD simulations using a force constant of 0.5 kcal (mol degree^2^)^−1^. For orientation-based BEUS simulations, the force constant for the ligand orientation angle (as in SMD simulations) was set to 0.5 kcal (mol degree^2^)^−1^. The force constant used for *r*_L_ and *r*_P_ in all cases was 1 kcal (mol Å^2^)^−1^. See Supplementary Fig. 1 for a schematic representation of these simulations.

### Free-energy calculations using non-parametric reweighting

Once the BEUS simulations described above were converged, a non-parametric reweighting method^76,78^, which is somewhat similar to the multi-state Bennett acceptance ratio method^79^, was used to construct the PMF. In this method^76^, each sampled configuration will be assigned a weight, which can be used to construct the PMF in terms of a desired collective variable. Suppose that a system is biased (for instance, within a BEUS scheme) using *N* different biasing potentials *U*_*i*_(**r**), where *i* = 1, …, *N*, and **r** represents all atomic coordinates. Typically, *U*_*i*_(**r**) is a harmonic potential defined in terms of a collective variable with varying centers for different *i*. Assuming an equal number of sampled configurations from each of the *N* generated trajectories, we can combine them in a single set of samples {**r**_*k*_} (irrespective of which bias was used to generate each sample **r**_*k*_) and determine the weight of each sample (*w*_*k*_) as:

32 $$w_k = c/\mathop {\sum}\limits_i {{\mathrm{e}}^{ - \frac{{\left( {U_i\left( {{{{\boldsymbol{r}}}}_k} \right) - F_i} \right)}}{{RT}}}}$$

where, *c* is the normalization constant such that $\mathop {\sum}\nolimits_k {w_k = 1}$ and both {*w*_*k*_} and perturbed free energies {*F*_*i*_} are determined iteratively using the above equation and the following:

33 $${\mathrm{e}}^{ - \beta F_i} = \mathop {\sum}\limits_k {w_k{\mathrm{e}}^{ - \frac{{U_i\left( {{{{\boldsymbol{r}}}}_k} \right)}}{{RT}}}}$$

Converged *w*_*k*_ values can be used to construct any ensemble averages including any PMF (for example, *G*(**ζ**) PMF of the atomic system in the collective variable space(**ζ**))) in terms of not only the collective variable used for biasing but also any other collective variables that are sufficiently sampled. One may use a weighted histogram method to construct the PMF as follows:

34 $$G\left( {{{{\boldsymbol{\upzeta }}}}_i} \right) = - RT\ln \mathop {\sum}\limits_k {w_k} \delta \left( {{{{\boldsymbol{\upzeta }}}}\left( {{{{\boldsymbol{r}}}}_k} \right) - {{{\boldsymbol{\upzeta }}}}_i} \right),$$

35 $$\delta \left( {{{{\boldsymbol{\upzeta }}}}\left( {{{{\boldsymbol{r}}}}_k} \right) - {{{\boldsymbol{\upzeta }}}}_i} \right) = \left\{ {\begin{array}{*{20}{l}} {1,} \hfill & {\left| {{{{\boldsymbol{\upzeta }}}}\left( {{{{\boldsymbol{r}}}}_k} \right) - {{{\boldsymbol{\upzeta }}}}_i} \right| < \left| {{{{\boldsymbol{\upzeta }}}}\left( {{{{\boldsymbol{r}}}}_k} \right) - {{{\boldsymbol{\upzeta }}}}_j} \right|{\mathrm{for}}\,j \ne i.} \hfill \\ {0,} \hfill & {{\mathrm{otherwise}}} \hfill \end{array}} \right.$$

To estimate the uncertainty of any of PMF calculations described above, one may use bootstrapping. Here, we have used a block Bayesian bootstrapping technique^77^, where 100 alternative datasets are resampled from the existing dataset and the same non-parametric reweighting algorithm and the same PMF calculation is repeated for each set to generate 100 alternative PMFs. The standard deviation of the PMF at any point along the reaction coordinate provides an estimate for the error.

### Alchemical FEP simulations

We used the BFEE2 (ref. 27) package to estimate the absolute free energy of binding in silico for an alchemical or geometrical route with multiple subprocesses and geometric constraints. Alchemical FEP simulations were performed to calculate the absolute binding free energy for the interaction of hFGF1 with heparin hexasaccharide. We used a double annihilation protocol^80^, wherein the heparin hexasaccharide is annihilated in both the free and bound states. The final conformations of the hFGF1–heparin complex^73^ and free heparin hexasaccharide equilibrium simulations (discussed previously in the ‘All-atom MD simulations’ section) were used to generate starting conformations for the bound hFGF1–heparin and free heparin FEP simulations respectively. For the alchemical route, four separate simulations are performed: (1) coupling the restraints of seven collective variables in the bound state; (2) decoupling the ligand alchemically in the bound state; (3) coupling the ligand alchemically in the unbound state; (4) decoupling the conformational restraints in the unbound state. The FEP simulations 1 and 3 were performed bidirectionally using 200 *λ*-windows (*λ* is the coupling parameter associated with the FEP that could vary between 0 and 1). Each *λ*-window included a 0.5 ns of equilibration and 5.0 ns of averaging for both the unbound and bound states, for a total of 2.3 μs (Supplementary Table 3). The decoupling FEP simulations 2 and 4 were also performed bidirectionally, each one for 51 ns. All FEP simulations were performed using the NAMD 2.13 (ref. 71) simulation package with the CHARMM36m all-atom additive force field, using the protocol discussed previously for the equilibrium simulations. We used the state-of-the-art BFEE2 (ref. 27) method to make input files and analyze the FEP simulations.

### Binding-free-energy calculations using geometrical route

The extended ABF technique with an umbrella integration estimator was used to calculate the free-energy change along the coarse variables required to characterize reversible heparin–hFGF1 binding^3,24,27^. We used the software BFEE2 (ref. 27) to generate the input files for these simulations. In the geometrical route, these collective variables are often subjected to restrictions, and the amount of reversible work required to impose each constraint is determined by a sequence of very accurate PMF simulations. The collective variables used here are the RMSDs of the two proteins’ backbone distances from the reference, native conformation, the three Euler angles (*Θ*, *Φ* and *Ψ*) that describe their relative orientation and the polar (*θ*) and azimuth angles (*φ*) that describe their relative position^27,81^. The geometrical path consists of a sequence of separate PMF computations performed sequentially with the gradual inclusion of restrictions (RMSD, *Θ*, *Φ*, *Ψ*, *θ* and *φ*), as shown in Supplementary Table 2. Each geometric collective variable (RMSD, Θ, *Φ*, Ψ, *θ*, *φ* and *r* = (1/*β*) ln(S*I*C°); *β* = (𝑘_B_*T*)^−1^, with *k*_B_ the Boltzmann constant and *T* the temperature; *C*° denotes the standard concentration of 1 M. I*, which stands for the separation term, and S*, which stands for the surface term, indicate the percentage of a sphere with radius r*, centered at the binding site of the reference protein, that is, accessible to its partner) simulation was run with 10 replicas per restriction, and each replica simulation included 20 ns (RMSD, *Θ*, *Φ*, *Ψ*, *θ* and *φ*) of simulation time (*r* collective variables simulations were run for 40 ns for each replica), for a total of 1.6 μs. The BFEE2 (ref. 27) Gui was used to analyze the final ABF simulation data.

### Reporting summary

Further information on research design is available in the Nature Portfolio Reporting Summary linked to this article.

### Supplementary information

Supplementary Information Supplementary Figs. 1–5 and Tables 1–3, providing additional data analysis.

Reporting Summary

### Source data

Source Data Fig. 1 Statistical source data.

Source Data Fig. 2 Statistical source data.

Source Data Fig. 3 Statistical source data.

Source Data Fig. 4 Statistical source data.

Source Data Fig. 5 Statistical source data.

## Data Availability

Datasets related to this article are deposited to the Zenodo repository^82^. Source data for Figs. 1–5 is available with this paper. Protein Data Bank (https://www.rcsb.org/) was used to collect the crystal structures2AXM ref. 69 and 1RG8 ref. 70.

## Code availability

All scripts as well as the full source code for non-parametric reweighting can be obtained from Zenodo^82^.
