# Supplementary material for: Binding affinity estimation from restrained umbrella sampling simulations
Source: Nat Comput Sci. 2022 Dec 29;3(1):59–70. doi: 10.1038/s43588-022-00389-9 (PMC10766565; doi:10.1038/s43588-022-00389-9)
Supplement: Supplementary file 1 — Supplementary Figs. 1–5 and Tables 1–3, providing additional data analysis. [file 43588_2022_389_MOESM1_ESM.pdf]

nature computational science

Article

https://doi.org/10.1038/s43588-022-00389-9

Binding affinity estimation from restrained umbrella sampling simulations

In the format provided by the authors and unedited

TABLE OF CONTENTS

SUPPLEMENTARY FIGURE 1 2

SUPPLEMENTARY TABLE 1 3

SUPPLEMENTARY TABLE 2 4

SUPPLEMENTARY TABLE 3 5

SUPPLEMENTARY FIGURE 2 6

SUPPLEMENTARY FIGURE 3 7

SUPPLEMENTARY FIGURE 4 8

SUPPLEMENTARY FIGURE 5 9

REFERENCE 10

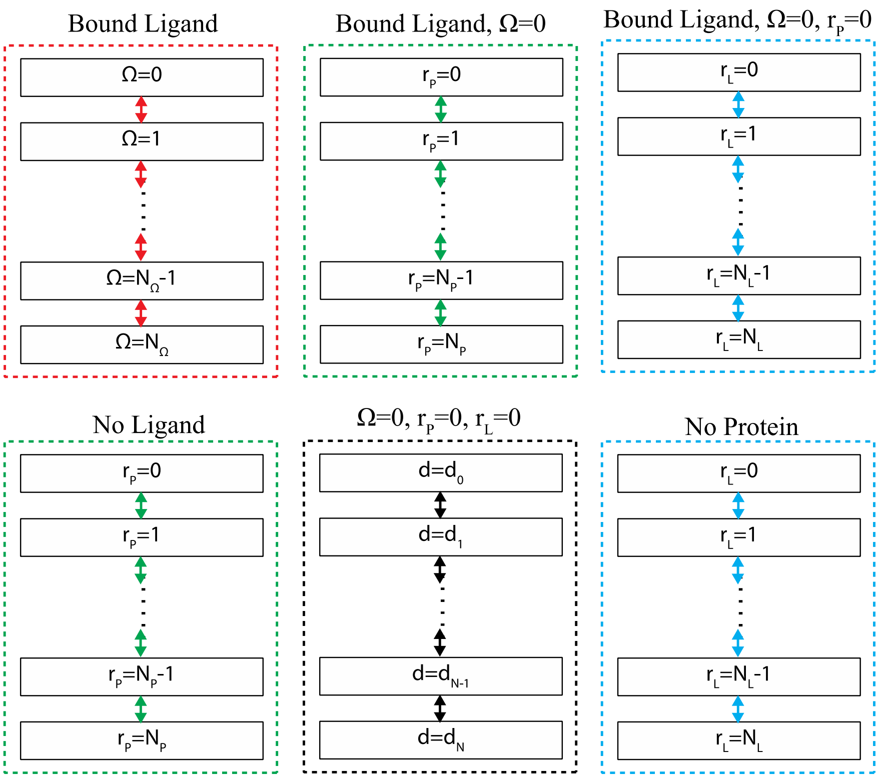

Supplementary Figure 1: Schematic illustration of one of the methods described in the manuscript that uses three types of restraints for PMF calculations. Schematic illustration of one of the methods described in the manuscript that uses three types of restraints for PMF calculations. Six independent sets of simulations are shown schematically in dashed boxes that were performed for our free energy calculations. Distance between the heavy-atom center of mass of heparin and that of the protein (d), the orientation angle of heparin with respect to the protein (Ω), RMSD of the protein (r_P) and RMSD of heparin (r_L) were used as collective variables. In the title of each simulation set, we have indicated the restraints used; e.g., $\Omega = 0$ indicates an orientation angle restraint on ligand. The individual boxes within each simulation set show the value of the collective variable in a specific unit system where 1 is equal to the distance between the centers of neighboring windows. The color of the box in above figure represents the varying collective variables red (Ω), green (r_P), blue (r_L), and black (d).

		No restraints	Ω restraint	r_L, r_P restraint	Ω, r_L, r_P restraint
Orientation correction (kcal/mol)	bulk<br>pocket	N/A	$9.9 \pm 0.0^*$<br>5.5 ± 0.3	N/A	9.9 ± 0.0<br>5.3 ± 0.3
Ligand RMSD correction (kcal/mol)	bulk<br>pocket	N/A	N/A	2.7 ± 0.1<br>2.1 ± 0.1	2.7 ± 0.1<br>2.1 ± 0.1
Protein RMSD correction (kcal/mol)	bulk<br>pocket	N/A	N/A	0.8 ± 0.1<br>0.5 ± 0.1	0.8 ± 0.1<br>0.5 ± 0.1
ΔG_V (kcal/mol)	bulk<br>pocket	4.4^{**}<br>0.7 ± 0.2	4.4<br>1.9 ± 0.2	4.4<br>2.1 ± 0.2	4.4<br>1.7 ± 0.2

Supplementary Table 1: Summary of the results of free energy calculations for bulk and pocket heparin-hFGF1 binding. This table represent the the ΔG_V of bluk and pocket using four different restraining methods along with correction terms. (*) All error estimates are based on one standard deviation (s.d.). ** This is an analytical value from relations 9 & 10 for ΔG_B . The orientation angle of heparin with respect to the protein (Ω), RMSD of the protein (r_P), RMSD of heparin (r_L), and the contribution of the difference between the volume of the binding pocket and the bulk to the binding free energy (ΔG_V) (see Theoretical foundation section in Methods for details). N/A means the data is not applicable in the corresponding section. We are comparing the ΔG_V of four different restraining methods (see Bias Exchange Umbrella Sampling (BEUS) simulations sub-section in Methods for details).

Absolute Binding Free Simulation	Number of Window	Simulation time for each window (ns)	PMF (kcal/mol)
ΔG(site,couple)*	200	5.5	-752.43 ± 0.81
ΔG(site,c+o+a+r)*	50	1	-14.36 ± 20.25
ΔG(bulk,decouple)*	200	5.5	739.92 ± 0.32
ΔG(bulk,c)*	50	1	15.92 ± 22.45
ΔG(bulk,o+a+r)	N/A		11.51
ΔG(total)	Total Simulation Time = 2302 ns		0.55 ± 30.25

Supplementary Table 2: Results showing the alchemical binding free-energy calculations of heparin-hFGF1 binding. This table represents the PMF values calculated for each step in FEP simulations along with simulation time and number of windows used in the simulations. **(*)** Alchemical free-energy calculations of the heparin binding FGF FEP simulations were also performed bi-directionally using 200 windows. Each window included a 0.5-ns equilibration and 5 ns of averaging for both the unbound and bound states, for a total of 2.3 µs (see Alchemical free energy perturbation (FEP) simulations sub-section in Methods for details).

Absolute Binding Free Simulation	Collective Variable	Restraints	Simulation time for each replica (ns)	Replicas	PMF (kcal/mol)
ΔG(site,c)*	RMSD	-	20	10	-10.45 ± 0.18
ΔG(site,eulerTheta)*	Θ	RMSD	20	10	-0.24 ± 0.01
ΔG(site,eulerPhi)*	Φ	RMSD, Θ	20	10	-0.22 ± 0.01
ΔG(site,eulerPsi)*	Ψ	RMSD, Θ, Φ	20	10	-0.31 ± 0.02
ΔG(site,polarTheta)*	θ	RMSD, Θ, Φ, Ψ	20	10	-0.60 ± 0.01
ΔG(site,polarPhi)*	φ	RMSD, Θ, Φ, Ψ, θ	20	10	-0.60 ± 0.02
(1/β)*ln(S*I*C0)*	r	RMSD, Θ, Φ, Ψ, θ, φ	40	10	-26.26 ± 2.81
ΔG(bulk,c)	RMSD	–			13.04 ± 0.07
ΔG(bulk,o)	–	–			6.61 ± 0.0
ΔG(total)			Total Simulation Time = 1600 ns		-19.04 ± 2.95

Supplementary Table 3: Results for each contribution to the binding free energy of the results of heparin-hFGF1 binding using ABF method. This table represents the PMF values calculated for each step in ABF simulations with simulation time and replicas used in method. (*) ABF calculations of the binding free energy for all the contribution of each degree of freedom. We followed free energy calculation stepwise strategy to the computation of the standard binding affinity of heparin binding FGF. The collective variables used here are the root-mean-square deviations (RMSDs) of the two proteins' backbone distances from the reference, native conformation, the three Euler angles (Θ, Φ, and Ψ) that describe their relative orientation and the polar (*θ*), azimuth angles (φ) that describe their relative position and r (r = (1/β)*ln(S*I*C°); $\beta=(k\mathrm{BT})^{-1}$, with kB the Boltzmann constant and T the temperature. C° denotes the standard concentration of 1M. I*, which stands for the separation term, and S*, which stands for the surface term, indicate the percentage of a sphere with radius r*, centered at the binding site of the reference protein, that is, accessible to its partner (see Binding free-energy calculations using geometrical route sub-section in Methods for details).

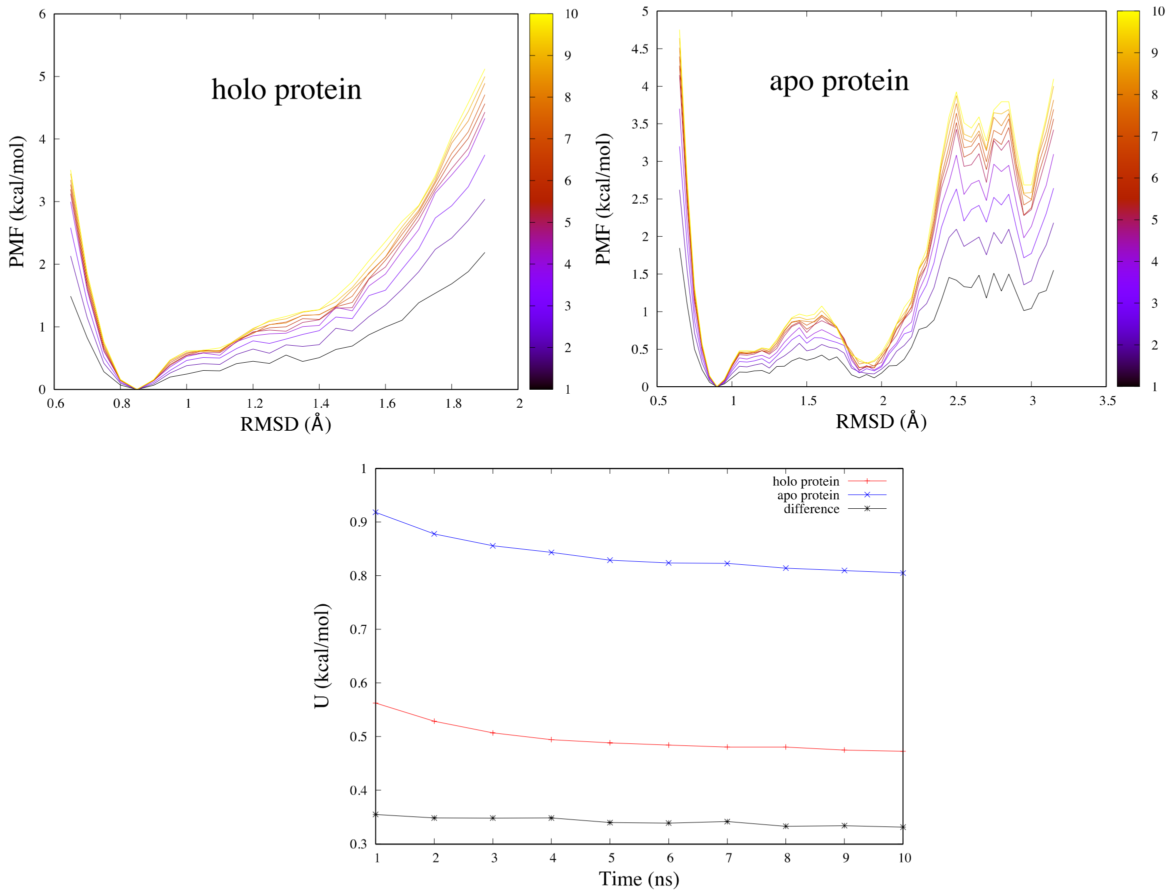

Supplementary Figure 2: Convergence Data for Protein RMSD PMFs. (Top) PMF calculated after 1, 2, …, 10 ns of BEUS simulations in terms of RMSD for apo (right) and holo (left) proteins. (Bottom) The correction term associated with protein RMSD restraint ($\Delta U_{r_P}^{r_L}$) as a function of simulation time.

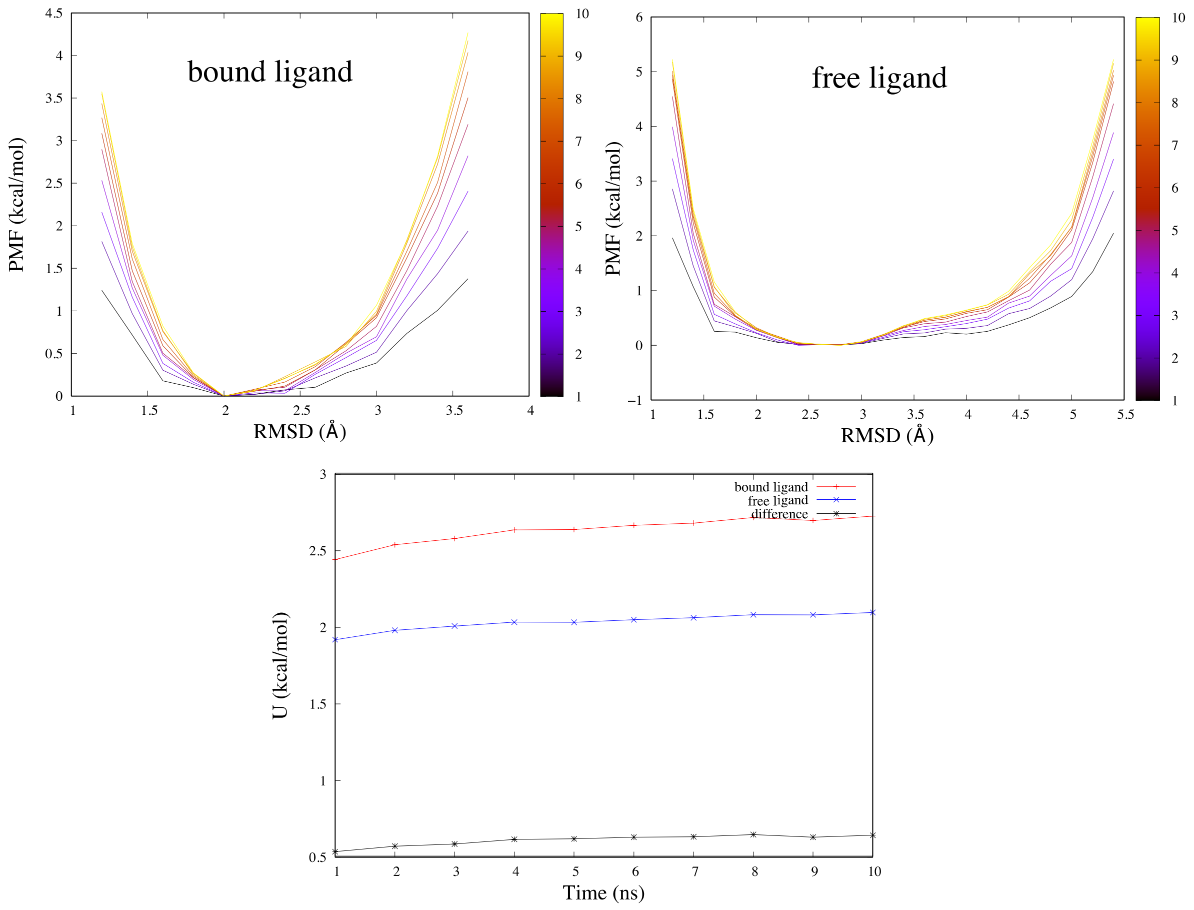

Supplementary Figure 3: Convergence Data for Ligand RMSD PMFs. (Top) PMF calculated after 1, 2, …, 10 ns of BEUS simulations in terms of RMSD for free (right) and bound (left) ligands. (Bottom) The correction term associated with ligand RMSD restraint (ΔU_{r_L}) as a function of simulation time.

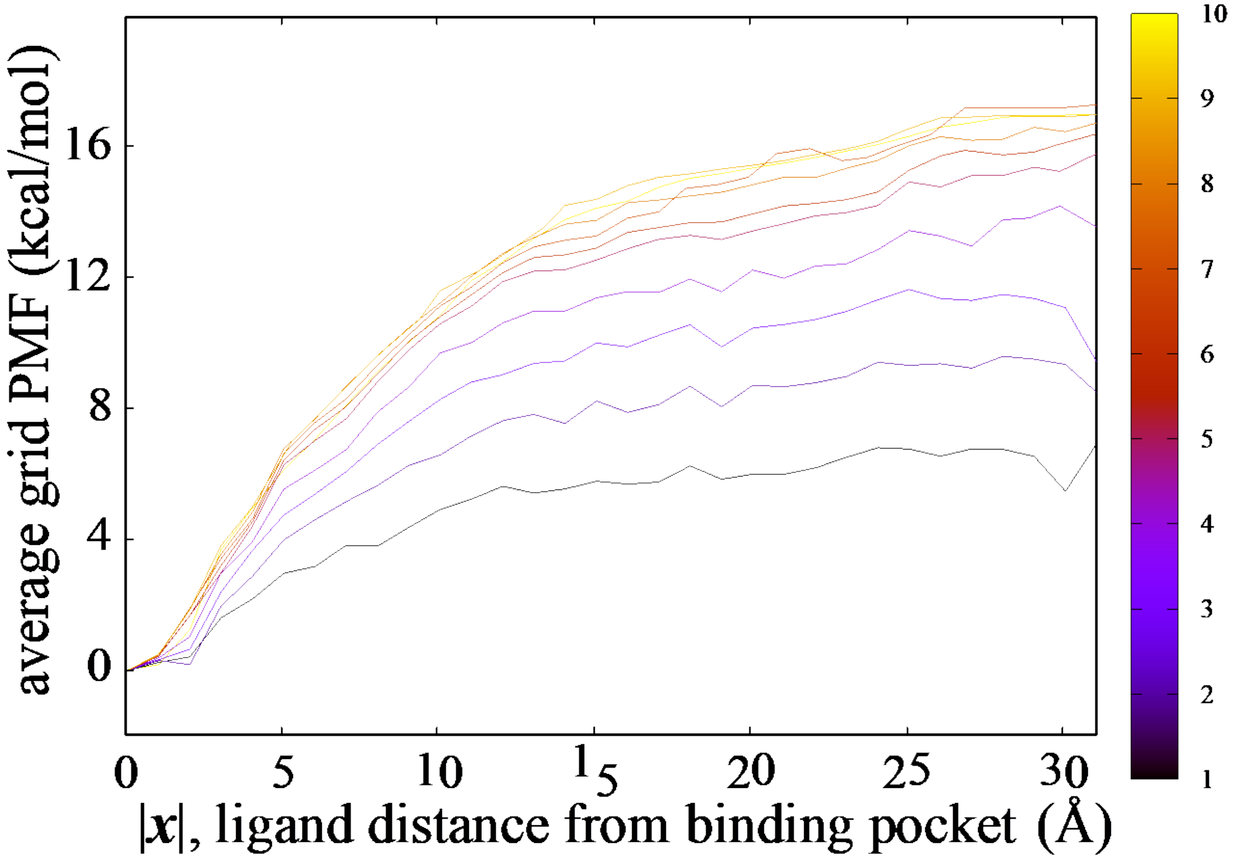

Supplementary Figure 4: Convergence Data for distance PMFs. PMF calculated after 1, 2, …, 10 ns of BEUS simulations in terms of ligand distance from binding pocket based on the distance-based BEUS simulation incorporating Ω, r_L, r_P restraints.

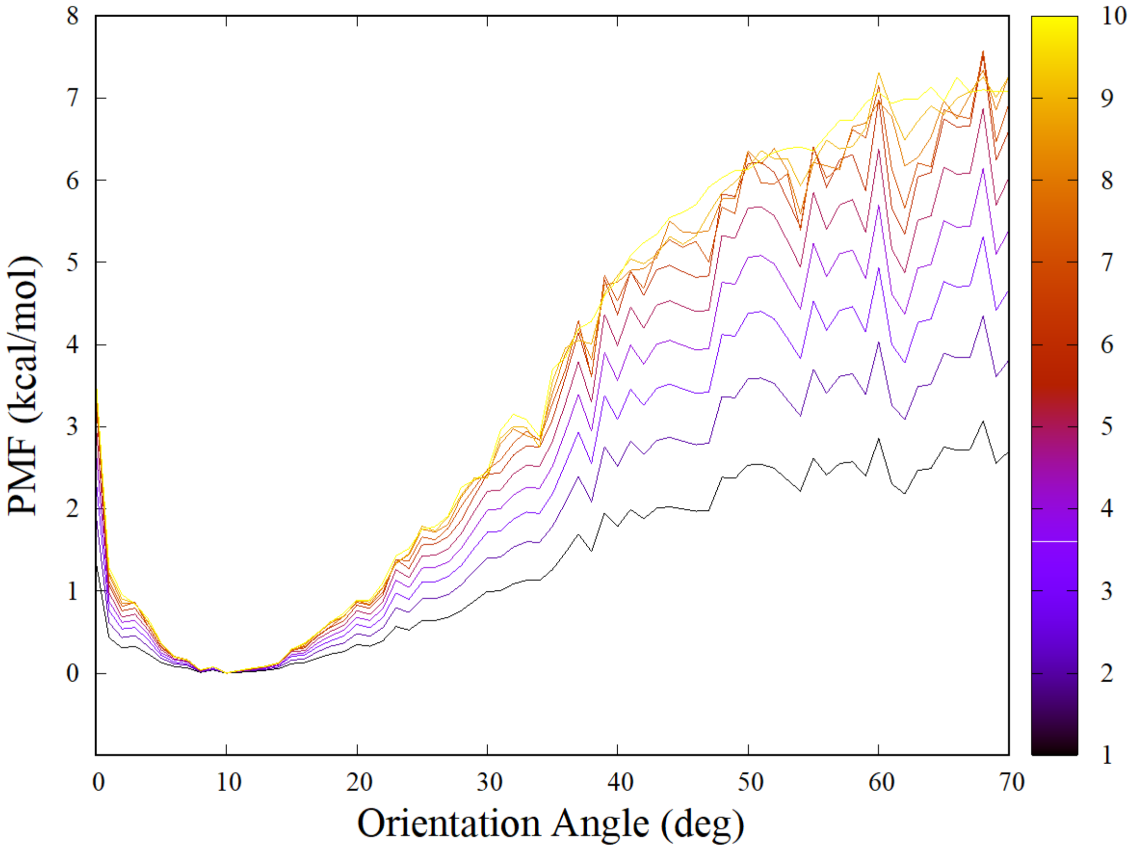

Supplementary Figure 5: Convergence Data for orientation PMFs. PMF calculated after 1, 2, …, 10 ns of BEUS simulations in terms of ligand orientation angle based on the orientation-based BEUS simulation incorporating no other restraints.

REFERENCES

1. Fu, H., Cai, W., Hénin, J., Roux, B. & Chipot, C. New Coarse Variables for the Accurate Determination of Standard Binding Free Energies. *J Chem Theory Comput* (2017) doi:10.1021/acs.jctc.7b00791.
2. Fu, H. *et al.* BFEE: A User-Friendly Graphical Interface Facilitating Absolute Binding Free-Energy Calculations. *J. Chem. Inf. Model* **58**, 556–560.
3. Fu, H., Chen, H., Cai, W., Shao, X. & Chipot, C. BFEE2: Automated, Streamlined, and Accurate Absolute Binding Free-Energy Calculations. *J Chem Inf Model* **61**, 2116–2123 (2021).
4. Woo, H.-J. & Roux, B. Calculation of absolute protein–ligand binding free energy from computer simulations. *Proc. Natl. Acad. Sci. U. S. A* **102**, 6825–6830.
