# Supplementary material for: Binding affinity estimation from restrained umbrella sampling simulations
Source: Nat Comput Sci. 2022 Dec 29;3(1):59–70. doi: 10.1038/s43588-022-00389-9 (PMC10766565; doi:10.1038/s43588-022-00389-9)
Supplement: Supplementary file 2 — Reporting Summary [file 43588_2022_389_MOESM2_ESM.pdf]

nature research

Corresponding author(s): Mahmoud Moradi

Last updated by author(s): Dec 2, 2022

Reporting Summary

Nature Research wishes to improve the reproducibility of the work that we publish. This form provides structure for consistency and transparency in reporting. For further information on Nature Research policies, see our Editorial Policies and the Editorial Policy Checklist.

Statistics

For all statistical analyses, confirm that the following items are present in the figure legend, table legend, main text, or Methods section.

n/a	Confirmed	
☐	☒	The exact sample size (*n*) for each experimental group/condition, given as a discrete number and unit of measurement
☐	☒	A statement on whether measurements were taken from distinct samples or whether the same sample was measured repeatedly
☐	☒	The statistical test(s) used AND whether they are one- or two-sided<br>*Only common tests should be described solely by name; describe more complex techniques in the Methods section.*
☒	☐	A description of all covariates tested
☒	☐	A description of any assumptions or corrections, such as tests of normality and adjustment for multiple comparisons
☐	☒	A full description of the statistical parameters including central tendency (e.g. means) or other basic estimates (e.g. regression coefficient) AND variation (e.g. standard deviation) or associated estimates of uncertainty (e.g. confidence intervals)
☒	☐	For null hypothesis testing, the test statistic (e.g. *F*, *t*, *r*) with confidence intervals, effect sizes, degrees of freedom and *P* value noted<br>*Give P values as exact values whenever suitable.*
☒	☐	For Bayesian analysis, information on the choice of priors and Markov chain Monte Carlo settings
☒	☐	For hierarchical and complex designs, identification of the appropriate level for tests and full reporting of outcomes
☒	☐	Estimates of effect sizes (e.g. Cohen's *d*, Pearson's *r*), indicating how they were calculated

Our web collection on statistics for biologists contains articles on many of the points above.

Software and code

Policy information about availability of computer code

Data collection	The ITC thermograms were analysed using Microcal origin software (Version # 7E). BFEE2 (version BFEE 2.3.1) was used to to generate input files for Alchemical free energy perturbation (FEP) simulations and Binding free-energy calculations using geometrical route simulations. NAMD 2.13 (version 2.13) was used to collect the molecular dynamics simulation data. Protein data bank (https://www.rcsb.org/) was used to collect the crystal structures 2AXM and 1RG8.
Data analysis	The ITC thermograms were analysed using Microcal origin software (Version # 7E). BFEE2 (version BFEE 2.3.1) was used to analyze Alchemical free energy perturbation (FEP) simulations and Binding free-energy calculations using geometrical route simulations data.

For manuscripts utilizing custom algorithms or software that are central to the research but not yet described in published literature, software must be made available to editors and reviewers. We strongly encourage code deposition in a community repository (e.g. GitHub). See the Nature Research guidelines for submitting code & software for further information.

Data

Policy information about availability of data

All manuscripts must include a data availability statement. This statement should provide the following information, where applicable:

- Accession codes, unique identifiers, or web links for publicly available datasets
- A list of figures that have associated raw data
- A description of any restrictions on data availability

Our simulations were based on the x-ray crystal structure of the dimeric complex with a heparin hexasaccharide (PDB:2AXM, resolution: 3.0 Å) and the model for the apo protein was based on the x-ray crystal structure of unbound monomeric hFGF1 (PDB: 1RG8, resolution: 1.1 Å). The list of figures that have the associated raw experimental data is Figure 4. There is no restriction on data availability from the ITC experiment.

Field-specific reporting

Please select the one below that is the best fit for your research. If you are not sure, read the appropriate sections before making your selection.

☒ Life sciences ☐ Behavioural & social sciences ☐ Ecological, evolutionary & environmental sciences

For a reference copy of the document with all sections, see nature.com/documents/nr-reporting-summary-flat.pdf

Life sciences study design

All studies must disclose on these points even when the disclosure is negative.

Sample size	ITC experiments were performed in triplicate under the same experimental conditions with the same sample. The variance in the ITC data, obtained under the same experimental conditions, is negligible. The triplicate simulations simply show that the measured binding affinities are highly reproducible under the same experimental conditions and estimated uncertainties are negligible. Therefore, the ITC experiments, conducted in triplicate is sufficient and n>3 would not provide additional information. For computational data associated with the new algorithm, the number of replicas are chosen based on the complexity of the problem to reach convergence, which is shown in supplementary figures 2-5.
Data exclusions	No datapoint in the ITC results were ommited. All datapoints in the isothermogram were used in analysis.
Replication	The isothermograms, obtained under the same experimental conditions, representing the heparin hexasaccharide - FGF1 titration were quite reproducible both qualitatively and quantitatively. ITC experiments were independently performed in triplicates and all the replications were successful.
Randomization	No experimental groups were used in this study. Randomization therefore was not applicable.
Blinding	The ITC experiment is a well-established experimental technique. Therefore, it was deemed that the blinding exercise was not necessary. No other experiment was performed to require blinding.

Reporting for specific materials, systems and methods

We require information from authors about some types of materials, experimental systems and methods used in many studies. Here, indicate whether each material, system or method listed is relevant to your study. If you are not sure if a list item applies to your research, read the appropriate section before selecting a response.

Materials & experimental systems

n/a	Involved in the study	
☒	☐	Antibodies
☒	☐	Eukaryotic cell lines
☒	☐	Palaeontology and archaeology
☒	☐	Animals and other organisms
☒	☐	Human research participants
☒	☐	Clinical data
☒	☐	Dual use research of concern

Methods

n/a	Involved in the study	
☒	☐	ChIP-seq
☒	☐	Flow cytometry
☒	☐	MRI-based neuroimaging
